# Quorum Sensing in Streptococcus mutans Regulates Production of Tryglysin, a Novel RaS-RiPP Antimicrobial Compound

**DOI:** 10.1128/mBio.02688-20

**Published:** 2021-03-16

**Authors:** Britta E. Rued, Brett C. Covington, Leah B. Bushin, Gabriella Szewczyk, Irina Laczkovich, Mohammad R. Seyedsayamdost, Michael J. Federle

**Affiliations:** a Department of Pharmaceutical Sciences, University of Illinois at Chicago, Chicago, Illinois, USA; b Department of Chemistry, Princeton University, Princeton, New Jersey, USA; KUMC

**Keywords:** *Streptococcus*, antimicrobial peptides, bacteriocins, quorum sensing

## Abstract

The genus *Streptococcus* encompasses a large bacterial taxon that commonly colonizes mucosal surfaces of vertebrates and is capable of disease etiologies originating from diverse body sites, including the respiratory, digestive, and reproductive tracts. Identifying new modes of treating infections is of increasing importance, as antibiotic resistance has escalated. Streptococcus mutans is an important opportunistic pathogen that is an agent of dental caries and is capable of systemic diseases such as endocarditis. As such, understanding how it regulates virulence and competes in the oral niche is a priority in developing strategies to defend from these pathogens. We determined that S. mutans UA159 possesses a bona fide short hydrophobic peptide (SHP)/Rgg quorum-sensing system that regulates a specialized biosynthetic operon featuring a radical-SAM (*S*-adenosyl-l-methionine) (RaS) enzyme and produces a ribosomally synthesized and posttranslationally modified peptide (RiPP). The pairing of SHP/Rgg regulatory systems with RaS biosynthetic operons is conserved across streptococci, and a locus similar to that in S. mutans is found in Streptococcus ferus, an oral streptococcus isolated from wild rats. We identified the RaS-RiPP product from this operon and solved its structure using a combination of analytical methods; we term these RiPPs tryglysin A and B for the unusual Trp-Gly-Lys linkage. We report that tryglysins specifically inhibit the growth of other streptococci, but not other Gram-positive bacteria such as Enterococcus faecalis or Lactococcus lactis. We predict that tryglysin is produced by S. mutans in its oral niche, thus inhibiting the growth of competing species, including several medically relevant streptococci.

## Abstract (precis)

Bacteria interact and compete with a large community of organisms in their natural environment. Streptococcus mutans is one such organism, and it is an important member of the oral microbiota. We found that S. mutans uses a quorum-sensing system to regulate production of a novel posttranslationally modified peptide capable of inhibiting growth of several streptococcal species.

## INTRODUCTION

In bacteria, quorum sensing (QS) coordinates a myriad of biological processes that include natural competence, sporulation, biofilm formation, and niche adaptation during host colonization in symbiotic and pathogenic interactions (1–3). Bacteria often use complex regulatory systems to sense and respond to QS signals termed “autoinducers” or “pheromones” (3–5). The chemical composition of autoinducers varies among species, but the best-characterized autoinducers are the signals of Gram-negative and Gram-positive bacteria that utilize acyl-homoserine lactones and oligopeptides, respectively (5, 6). In recent years, a class of transcriptional regulators that respond to peptide-based QS signals has emerged as important regulatory factors in Gram-positive species: the RRNPP (Rap/Rgg/NprR/PlcR/PrgX) family (7, 8). Among members of this family, the Rgg regulators are the most common subclass encoded in streptococci, and several occurrences of Rgg paralogs are frequently observed within any given genome. Rgg regulators sense and respond to SHPs (short hydrophobic peptides) by directly binding these linear peptides, which induce allosteric changes in Rgg transcriptional activity (7, 9–16). Rgg proteins have been characterized in various streptococcal species, including the medically relevant Streptococcus pyogenes (17, 18), Streptococcus pneumoniae (11–13), and Streptococcus mutans (19–21). This family of proteins were originally identified in Streptococcus gordonii, as regulators of glucosyltransferase activity, and as such were named Rgg for regulator gene of glucosyltransferase (22, 23). Multiple studies demonstrate their involvement in processes such as virulence gene regulation, biofilm formation, capsule production, resistance to host factors, and competence (9–15, 19, 20, 24).

SHP/Rgg systems are frequently associated with RaS-RiPP biosynthetic operons in streptococci (25, 26). RaS (radical *S*-adenosyl-l-methonine)-RiPP (ribosomally synthesized and posttranslationally modified peptides) operons give rise to peptide secondary metabolites that are modified by one or more RaS enzymes, a large enzyme superfamily characterized by its ability to reductively activate SAM (*S*-adenosyl-l-methionine) with the aid of an active site-bound [4Fe-4S] cluster (27, 28). RaS-RiPP operons typically code for a precursor peptide, a RaS enzyme, in some cases an RRE (RiPP recognition element) protein, and additional genes for modification and transport (26, 29, 30). The RaS enzymes in these operons install unprecedented cyclization motifs onto the precursor peptides via a variety of biochemical reactions (25, 26, 31–33). However, while the reactions carried out by the RaS enzymes in several RaS-RiPP operons have been characterized, the final secondary metabolite is in most cases unknown, and the biological relevance of these peptides remains unexplored (34).

This report focuses on an SHP/Rgg system linked to the “WGK” RaS-RiPP operon, named for a conserved Trp-Gly-Lys motif in the precursor peptide. Previous work determined that the RaS enzyme in this cluster catalyzes a unique modification involving a four-electron oxidation in a single step (26). The mature product and its role in the physiology of its hosts, including S. mutans and Streptococcus ferus, are not known. S. mutans is an important factor in the development of dental caries and a causative agent of infective endocarditis (35, 36). The high prevalence of S. mutans in dental plaque is associated with increased propensity for caries (37). S. mutans does not exist singularly in these plaques, however, and interacts with many other microorganisms (36, 38, 39). To compete in the oral niche, S. mutans makes use of QS-regulated bacteriocins, generally referred to as mutacins, that inhibit growth of competing species (35, 38, 40, 41, 42). Less is known about S. ferus; it is also a member of the mutans group of streptococci and was originally isolated from wild rodents (43) but also found to colonize the oral cavity of pigs (44). Reactions of enzymes encoded in the WGK cluster were previously elucidated in S. ferus (26). In this study, we characterize the product of the WGK RaS-RiPP operon, a novel secondary metabolite that we call tryglysin A (from *S. ferus*) and tryglysin B (from S. mutans). The modified peptides possess inhibitory activities reminiscent of nonlantibiotic mutacins. We demonstrate that the identified SHP/Rgg system directly regulates tryglysin production in S. mutans and that this peptide is likely important for competition with other organisms in its dental niche.

## RESULTS

### Identification of an SHP/Rgg signaling system paired to an operon producing a RaS-RiPP in S. mutans UA159.

In a recent publication from one of our laboratories, it was determined that a large RaS-RiPP SHP/Rgg network exists in streptococci (26). It was found that the identified RaS-RiPP systems produce a variety of posttranslationally modified peptides, and the structures of several classes of these peptides were determined (26, 31, 32). While the biochemical reactions of the RaS-RiPP systems that produce these peptides were characterized, we have yet to empirically test their physiological relevance and linkage to SHP/Rgg systems in streptococci. To begin examining the connection of Rgg transcriptional regulators to RaS-RiPP systems, we started with a system in a well-studied and genetically tractable organism, Streptococcus mutans UA159.

**FIG 1:**
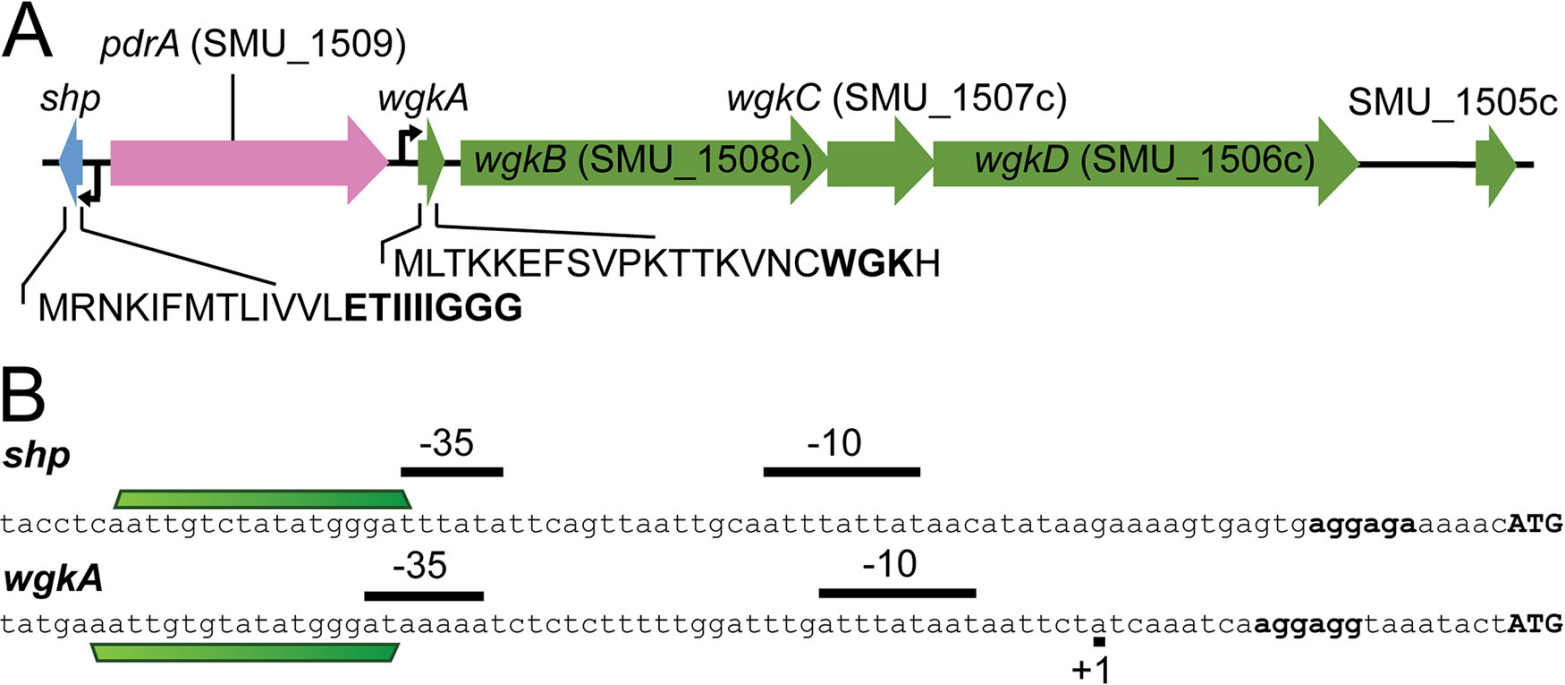
The genomic context of the PdrA (SMU_1509) system in S. mutans UA159. (A) Schematic depicting the genetic locus of the signaling system. The SHP signaling peptide and *pdrA* (SMU_1509) are transcribed divergently as illustrated (blue and pink arrows, respectively). The full-length peptide sequence for SHP is indicated below the graphical representation, with the active version (SHP) used for induction shown in bold type. The *wgk* operon is encoded following *pdrA*, as indicated by green arrows. The *wgk* operon consists of *wgkA*, *wgkB* (SMU_1508c), *wgkC* (SMU_1507c), *wgkD* (SMU_1506c), and SMU_1505c. The WGK precursor peptide sequence is indicated below *wgkA*, with the previously identified modified sequence (WGK) shown in bold type. (B) Promoter architecture of the *shp* and *wgkA* promoters. The −10 and −35 elements are illustrated by black bars, as predicted by BPROM or experimentally determined by 5′ RACE. The transcription start site as determined by 5′ RACE for the *wgkA* promoter is illustrated by the +1 symbol. The conserved motif, AATTGT**X**TATATGGGAT, in the *shp* and *wgkA* promoters is indicated by the green trapezoid. The conserved motif was determined by nucleotide alignment with Clustal Omega.

S. mutans UA159 contains a WGK RaS-RiPP system orthologous to that of *S. ferus*, for which the biochemical reaction of the RaS enzyme was recently elucidated (26). The system in S. mutans includes an Rgg regulator (SMU_1509, henceforth referred to as PdrA, for pheromone-dependent regulator of RiPP) and an adjacent SHP-encoding gene (Fig. 1A). The RaS-RiPP genes include *wgkA*, a previously unannotated sequence encoding a 21-amino-acid prepeptide, *wgkB* (SMU_1508c), a RaS enzyme, *wgkC* (SMU_1507c), coding for an RRE protein, *wgkD* (SMU_1506c), a predicted transporter, and SMU_1505c, a pseudogene with homology to the beta-subunit of phenylalanine-tRNA ligase (Fig. 1A). The WGK peptide of S. mutans is predicted to be similar in its modification site and sequence to WGK from *S. ferus*, with the exception of the replacement of a serine residue with a cysteine at amino acid 17 in the prepeptide (Fig. 1A) (26). This operon appears to be part of the S. mutans core genome, as a nucleotide BLAST search indicated that PdrA and the adjacent *wgk* operon are conserved (greater than 99% identity) in 19 strains of S. mutans deposited in GenBank (Table 1).

**TABLE 1:** Conservation of *pdrA*, the *wgk* operon, and the putative PdrA binding sequence in Streptococcus mutans^*a*^

Strain	% identity	Conservation of PdrA binding sequence^*b*^
UA159	100	Yes
UA159-FR	100	Yes
LAB761	99.98	Yes
KCOM 1054	99.96	Yes
MD	99.89	Yes
NCTC10832	99.89	Yes
P1	99.82	Yes
P6	99.82	Yes
S1	99.82	Yes
S4	99.82	Yes
FDAARGOS_685	99.69	Yes
NBRC 13955	99.69	Yes
NCTC10449	99.69	Yes
LJ23	99.62	Yes
NN2025	99.60	Yes
GS-5	99.58	Yes
LAR01	99.51	Yes
T8	99.37	Yes
UA140	99.20	Yes

a Compared to S. mutans UA159 sequence of *pdrA* and the *wgk* operon by nucleotide BLAST. For further details concerning analysis, see Materials and Methods.

b Conservation of the predicted PdrA binding sequence, AATTGT**X**TATATGGGAT (see Fig. 1B and text for details).

Orthologs of PdrA and the adjacent SHP peptide are documented to closely resemble Rgg systems in Streptococcus agalactiae, Streptococcus thermophilus, and S. pyogenes, and an active form of SHP (ETIIIIGGG) (Table 2) has been reported (9, 45). However, regulatory function of PdrA on the proximal RaS-RiPP system has not been tested in S. mutans. As Rgg regulators typically bind near canonical promoter elements, we wondered whether the promoter architecture of the *wgk* operon and *shp* gene possessed a potential Rgg binding motif. Clustal Omega was used to perform a basic nucleotide alignment of the promoters predicted by the online algorithm BPROM (46, 47). The transcription start site (TSS) of the *wgk* operon was determined by rapid amplification of cDNA ends (5′ RACE), but attempts to map the TSS of *shp* were unsuccessful (Fig. 1B). The predicted −35 and −10 elements from the BPROM algorithm closely aligned to the experimentally determined transcription start of *wgk*. Upon comparison of the promoter architectures, we also observed a conserved sequence near the predicted −35 element of both the *shp* and *wgk* promoters (Fig. 1B). The conserved sequence, AATTGT**X**TATATGGGAT, is 17 nucleotides in length, and we predict this to be the binding sequence for PdrA (Fig. 1B). This sequence is completely conserved in S. mutans strains containing the *pdrA*/*wgk* locus (Table 1).

**TABLE 2:** Amino acid sequences of peptides used in this publication^*a*^

Peptide	Amino acid sequence
SHP	ETIIIIGGG
Reverse SHP	GGGIIIITE
Tryglysin A	VNSWGKH
Tryglysin B	VNCWGKH
XIP	GLDWWSL

a SHP and reverse SHP were synthesized by ABClonal, tryglysins A and B were synthesized in-house, and XIP was synthesized by NeoScientific. See Materials and Methods for additional details.

### PdrA and its cognate SHP constitute an active SHP/Rgg system in S. mutans.

To determine whether SHP was able to induce the Rgg system, we constructed a luciferase reporter in which the putative promoter of *shp* (P*_shp_*) was linked to *luxAB* in the *galK* locus of the S. mutans chromosome (*galK*::P*_shp_-luxAB*-P_c_-*erm*, BRSM69, Table S1). The *gal* locus was previously reported to be nonessential in a transposon sequencing (Tn-seq) study of S. mutans UA159 in both *in vitro* and *in vivo* conditions (48) and is not required for growth in glucose, although it is essential for growth on galactose exclusively (49). No growth defects were observed for luciferase reporter strains grown in peptide-free, chemically defined medium (CDM) containing 1% glucose (see Fig. S1A in the supplemental material); reporters generated at the *galK* locus were therefore used in subsequent assays.

10.1128/mBio.02688-20.1 TABLE S1 Bacterial strains, plasmids, and primers used in this study. Download Table S1, PDF file, 0.3 MB. Copyright © 2021 Rued et al. 2021 Rued et al. https://creativecommons.org/licenses/by/4.0/ This content is distributed under the terms of the Creative Commons Attribution 4.0 International license.

10.1128/mBio.02688-20.3 FIG S1 Strain UA159 does not have a growth defect in CDM when luciferase reporters are inserted into the *galK* locus. UA159 has a small increase in doubling time upon SHP addition. These experiments were performed a minimum of two times with similar results. (A) Growth curve of wild-type UA159, *galK:*:P*_shp_-luxAB*-P_c_-*erm* (P*_shp_-luxAB*), and *galK:*:P*_wgkA_-luxAB*-P_c_-*erm* (P*_wgkA_-luxAB*) strains in CDM. (B) Growth curve of wild-type UA159, *galK:*:P*_shp_-luxAB*-P_c_-*erm* (P*_shp_-luxAB*), and *galK:*:P*_wgkA_-luxAB*-P_c_-*erm* (P*_wgkA_-luxAB*) in the presence of increasing concentrations of SHP or reverse SHP (revSHP). Average doubling times for each strain and condition are indicated in minutes. Means ± standard errors of the means (SEM) are shown. Download FIG S1, PDF file, 0.2 MB. Copyright © 2021 Rued et al. 2021 Rued et al. https://creativecommons.org/licenses/by/4.0/ This content is distributed under the terms of the Creative Commons Attribution 4.0 International license.

**FIG 2:**
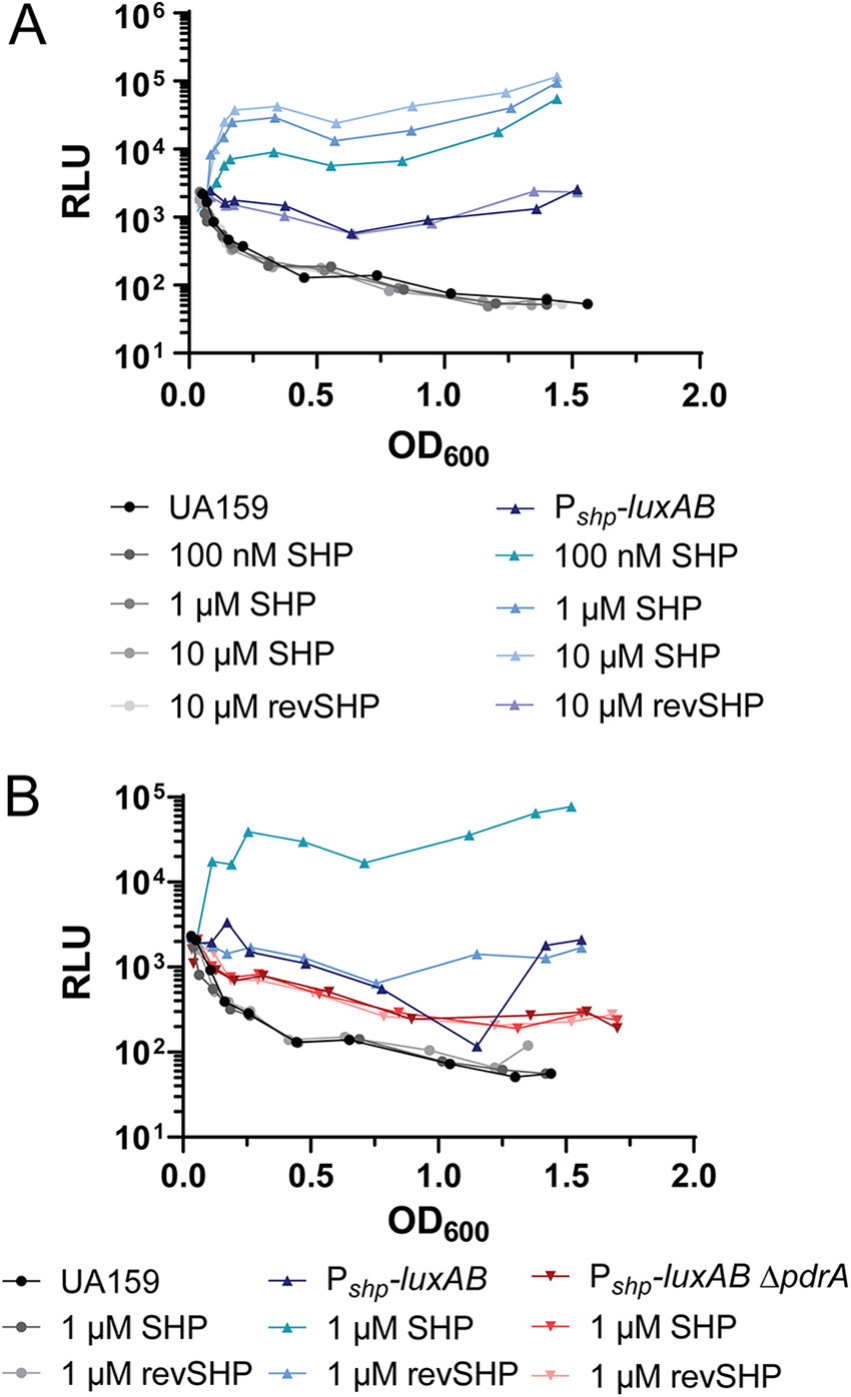
The identified Rgg system in S. mutans UA159 constitutes an active signaling system. (A) P*_shp_* is induced by the addition of SHP peptide in a dose-dependent manner. Wild-type UA159 and *galK:*:P*_shp_-luxAB*-P_c_-*erm* (P*_shp_-luxAB*) strains were incubated with increasing concentrations of SHP peptide (no peptide, 100 nM, 1 μM, 10 μM) or 10 μM reverse SHP (revSHP) peptide as a negative control. RLU, relative light units. (B) PdrA is required for induction of the P*_shp_* promoter response to SHP. Wild-type UA159, *galK:*:P*_shp_-luxAB*-P_c_-*erm* (P*_shp_-luxAB*), and *galK:*:P*_shp_-luxAB*- P_c_-*erm* Δ*pdrA*::*spec* (P*_shp_-luxAB* Δ*pdrA*) strains were incubated without SHP, with 1 μM SHP, or with 1 μM reverse SHP as a negative control. These experiments were performed a minimum of two times with similar results.

We first examined whether synthetic SHP was able to induce the P*_shp_-luxAB* reporter and found that upon titration of increasing amounts of SHP, a proportionate increase in luciferase activity was observed (Fig. 2A). This response appeared to be maximal at 1 to 10 μM SHP (Fig. 2A). As a negative control, a peptide in which the amino acid sequence of SHP was reversed (reverse SHP, Table 2) did not induce a response in luciferase activity at a concentration of 10 μM (Fig. 2A). Cultures of the P*_shp_-luxAB* reporter without exogenously supplied SHP displayed approximately 3- to 10-fold-higher luminescence values than cultures of UA159 containing no reporter, suggesting a basal level of *shp* transcription occurs (Fig. 2A). It is notable that a slight impact on growth of strain UA159 occurred upon addition of SHP peptide (Fig. S1B). In contrast, no increase in doubling time was observed with the reversed-sequence SHP peptide. A deletion mutation of *pdrA*, in which the *pdrA* coding sequence was replaced with a spectinomycin cassette (see Table S1 in the supplemental material), was used to examine any possible SHP-dependent responses. Addition of 1 μM SHP or reverse SHP led to no observable induction of luciferase activity (Fig. 2B). Overall, these results demonstrate that SHP induces a dose- and Rgg-dependent response of the *shp* promoter.

As other Rgg systems have demonstrated the requirement of the PptAB peptide exporter and the Opp oligopeptide permease importer (9, 10, 19, 50, 51), we generated mutants of the corresponding *pptAB* and *oppD* genes in S. mutans and transferred a plasmid-based reporter of P*_shp_-luxAB* into them. Δ*pptAB* strains remained responsive to the addition of SHP peptide, although they reached lower activity levels than wild-type strains (Fig. S2A). This result is expected if export of natively produced SHP contributes to the induction of the system, i.e., *pptAB* deletion disrupts the SHP autoinduction loop observed in other Rgg systems. In contrast, we observed that Δ*oppD* strains were unresponsive to SHP peptide (Fig. S2B). This was also expected, as deletion of the peptide importer should abrogate the ability of both synthetic and natively produced SHP peptides to enter cells. This result also indicates that SHP is unable to bypass the export/import system, a phenomenon observed in the ComR/XIP signaling system of S. mutans (19, 20, 52, 53).

10.1128/mBio.02688-20.4 FIG S2 Additional luciferase assays examining the requirement of the PptAB exporter and Opp importer for PdrA/SHP signaling and whether cross talk occurs between the ComR/XIP and PdrA/SHP systems. (A) The PptAB exporter is important for the PdrA/SHP signaling system. Luciferase activity was measured over time in CDM after the addition of 10 μm SHP or equivalent volume of DMSO carrier to a plasmid-based P*_wgkA_-luxAB* reporter in wild-type S. mutans UA159 or Δ*pptAB*::*aphA3* strain. This experiment was performed three times in triplicate. (B) The Opp importer is important for the PdrA/SHP signaling system. Luciferase assay of a plasmid-based P*_wgkA_-luxAB* reporter in wild-type S. mutans UA159 or Δ*oppD*::*cat* strain. This experiment was performed as outlined above for panel A and performed three times in triplicate. (C) Luciferase assay of a chromosome-based P*_shp_-luxAB* reporter in wild-type S. mutans UA159 with the addition of 1 μM XIP, SHP, or reverse SHP (revSHP) peptides. This experiment was performed twice. (D) Luciferase assay of a plasmid-based P*_sigX_-luxAB* reporter in wild-type S. mutans UA159 with the addition of 1 μM XIP, SHP, or reverse SHP (revSHP) peptides. This experiment was performed twice. Download FIG S2, PDF file, 0.3 MB. Copyright © 2021 Rued et al. 2021 Rued et al. https://creativecommons.org/licenses/by/4.0/ This content is distributed under the terms of the Creative Commons Attribution 4.0 International license.

We additionally examined whether cross talk between the ComR/XIP (*comX*-inducing peptide) signaling system and the newly identified PdrA/SHP system could occur. Previous studies have demonstrated that cross talk occurs between Rgg-like regulators within strains and between different species of streptococci (13, 45, 54–56). We took cultures of the P*_shp_-luxAB* reporter and a plasmid-based P*_sigX_-luxAB* reporter and examined whether they could be induced by addition of either the noncognate XIP or SHP peptide (Fig. S2C and D). We did not observe any induction of the P*_shp_-luxAB* reporter to XIP or the P*_sigX_-luxAB* reporter to SHP peptide (Fig. S2C and D). Therefore, we conclude that at least between the PdrA/SHP system and the ComR/XIP signaling system in S. mutans, cross talk does not appear to occur.

### Induction of the identified SHP/Rgg system affects transcription of multiple targets in S. mutans.

**FIG 3:**
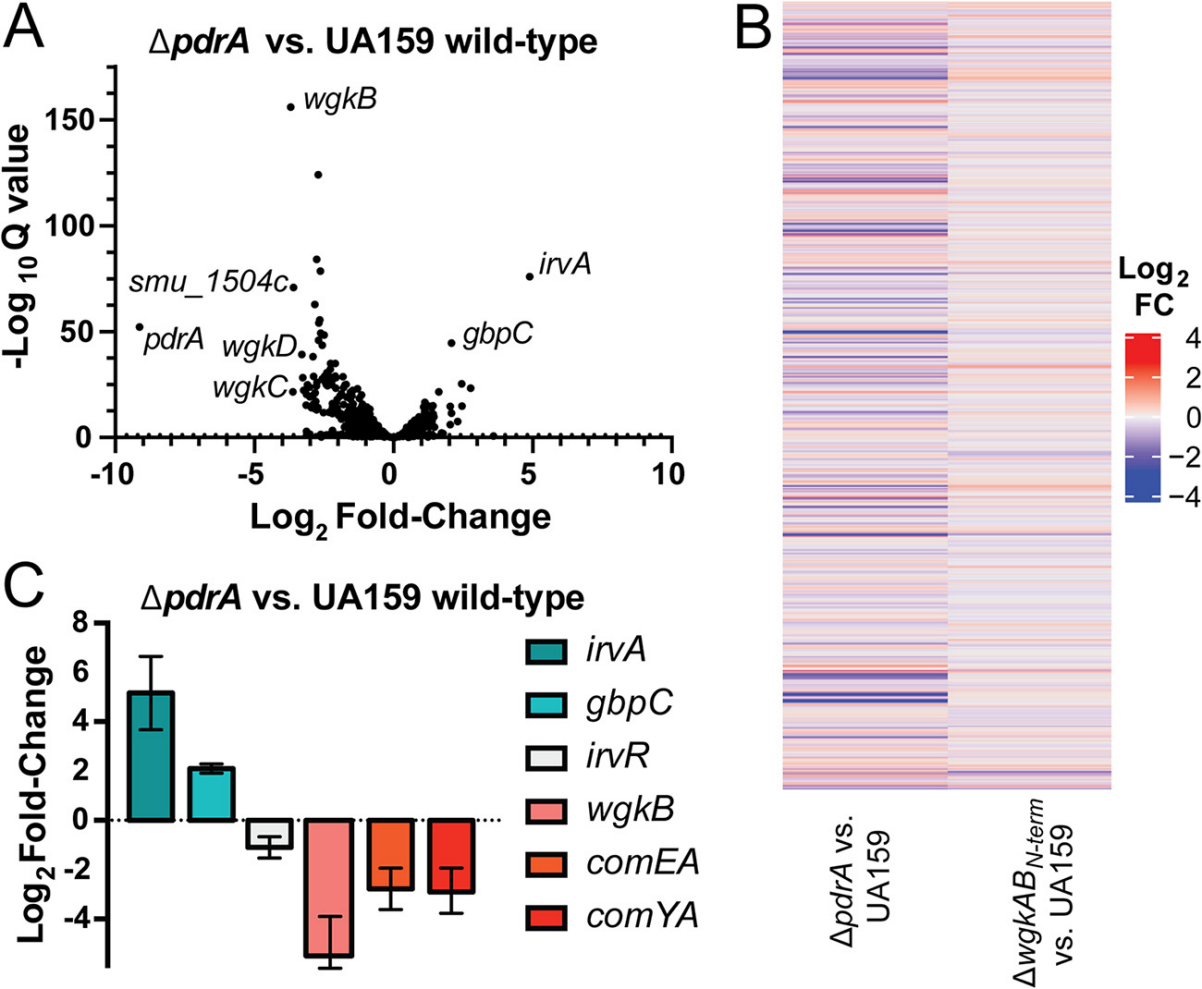
RNA-seq identifies genes regulated by the PdrA system in the presence of SHP. (A) Volcano plot of Δ*pdrA* versus wild-type UA159 transcript fold change upon the addition of SHP. Δ*pdrA*::*spec* and wild-type UA159 were grown to an OD_600_ of 0.17 to 0.2 and induced with 10 μM SHP. RNA was collected mid-exponential phase and subsequently processed for RNA-seq. Genes of interest are indicated on the graph. For a complete list of differentially regulated genes and RNA-seq data, see Data Set S1 in the supplemental material. (B) Heatmaps indicating differentially regulated transcripts in the UA159 genome upon deletion of *pdrA* (SMU_1509) or *wgkAB*_N-term_ (*wgkA* and SMU_1508c) versus wild-type UA159 in the presence of 10 μM SHP. Increasing red color indicates increased log_2_ fold change (log_2_FC) in transcripts, whereas increasing blue color indicates decreased log_2_ fold change in transcripts. (C) qRT-PCR validation of select targets from RNA-seq. Δ*pdrA*::*spec* and wild-type UA159 were treated with 10 μM SHP, and relative transcript levels were compared. Genes are indicated according to color and legend beside graph. Values are means ± standard deviation (error bars) of the gene transcript levels measured from three independent experiments performed in triplicate.

To determine the genome-wide transcriptional impact of the PdrA system, Illumina RNA sequencing (RNA-seq) was performed on exponential-growth-phase cultures of wild-type and Δ*pdrA* S. mutans strains treated with 10 μM SHP. Sequencing results determined that 81 transcripts were differentially expressed (log_2_ fold change > 2, Q-value < 0.5; Fig. 3A and B, left column). As expected, count values of the *pdrA* transcript of the deletion strain were at background levels (Fig. 3A). Strikingly, the Δ*pdrA* strain displayed lowered transcription of genes in the *wgk* operon: *wgkA*, *wgkB*, *wgkC*, and *wgkD* (Fig. 3A; also see Data Set S1 in the supplemental material). Other gene transcripts observed to be lower in the Δ*pdrA* strain included those involved in competence (*comEA*, *comYA*) and metabolism (*ackA*, *pdhC*). Genes seen as more highly expressed in the Δ*pdrA* strain included factors previously found to be important for adhesion of S. mutans to the tooth surface and implicated in virulence, *gbpC* and *irvA* (Fig. 3A) (57–59). A complete list of differentially regulated genes, normalized expression data, and statistics are provided in the Data Set S1 file.

10.1128/mBio.02688-20.9 DATA SET S1 RNA-seq data and differential expression analysis. Download Data Set S1, XLSX file, 0.9 MB. Copyright © 2021 Rued et al. 2021 Rued et al. https://creativecommons.org/licenses/by/4.0/ This content is distributed under the terms of the Creative Commons Attribution 4.0 International license.

To validate results of the RNA-seq profiles, cultures of Δ*pdrA* and wild-type strains were again grown to early exponential growth phase, and RNA was extracted 60 min after treatment with 10 μM SHP. Quantitative reverse transcription-PCR (qRT-PCR) was conducted on transcripts of *irvA*, *gbpC*, *wgkB*, *comEA*, and *comYA*, as well as *irvR*, as the latter constitutes part of the IrvA/GbpC regulatory circuit (58). Results confirmed that the Δ*pdrA* strain produced higher levels of *irvA* and *gbpC*, while *wgkB*, *comEA*, and *comYA* were downregulated compared to wild type (Fig. 3C and Data Set S1). No statistically significant difference was seen for the *irvR* transcript between strains; therefore, changes observed in *irvA* and *gbpC* levels are not likely to involve IrvR (Fig. 3C and Data Set S1).

To test the possibility that the WGK peptide acts as part of a signaling system within S. mutans, a strain (Δ*wgkAB*_N-term_) was generated that deletes the entire *wgkA* reading frame and the first 992 nucleotides of *wgkB* in strain UA159. Transcriptomic analysis using RNA sequencing was conducted for Δ*wgkAB*_N-term_ in parallel with the UA159 and Δ*pdrA* samples described above including treatment with 10 μM SHP to assure expression of the *wgk* operon. Overall, changes in global transcription were not observed to be significant, except for decreased expression of the *wgk* operon, likely caused by polar effects of the generated deletion in *wgkAB*, and a modest increase in the level of *gbpC* (Fig. 3B, right column; see also Fig. S3A and B and Data Set S1). Overall, we conclude that WGK operon does not function as a regulatory system or have direct impact on transcriptional programs in S. mutans under these conditions.

10.1128/mBio.02688-20.5 FIG S3 RNA-seq and HR-MS/MS data for tryglysins. (A) RNA-seq identifies genes differentially regulated upon deletion of the tryglysin B precursor peptide. Volcano plot of Δ*wgkAB_N-term_* versus wild-type UA159 transcript fold change. Δ*wgkAB_N_* and wild-type UA159 were grown to an OD_600_ of 0.17 to 0.2 and induced with 10 μM SHP. RNA was collected mid-exponential phase and subsequently processed for RNA-seq. The *wgk* operon and other genes of interest are indicated. (B) Volcano plot of Δ*wgkAB_N-term_* versus Δ*pdrA* transcript fold change. Strains were induced with SHP and harvested for RNA as described for panel A. Genes of interest are indicated on the graph. For a complete list of differentially regulated genes from experiments in panels A and B, see Data Set S1. (C and D) HR-MS/MS analysis of authentic tryglysin A from *S. ferus* (C) and synthetic tryglysin A (D). Some fragments are marked. See Table S2A to D for further information. The analogous parent/fragment HR-MS data and chromatographic behavior show that these molecules are identical. Download FIG S3, PDF file, 0.2 MB. Copyright © 2021 Rued et al. 2021 Rued et al. https://creativecommons.org/licenses/by/4.0/ This content is distributed under the terms of the Creative Commons Attribution 4.0 International license.

10.1128/mBio.02688-20.2 TABLE S2 (A) HR-MS and HR-MS/MS data for authentic tryglysin A from *S. ferus* DSM 20646. (B) HR-MS data for synthetic tryglysin A and B. (C) HR-MS/MS data for synthetic tryglysin A. (D) HR-MS data for modified HRV-3C-cleaved WgkA peptides, intermediates for the preparation of synthetic tryglysin A and B. (E) HR- MS/MS data for synthetic tryglysin B. Download Table S2, PDF file, 0.2 MB. Copyright © 2021 Rued et al. 2021 Rued et al. https://creativecommons.org/licenses/by/4.0/ This content is distributed under the terms of the Creative Commons Attribution 4.0 International license.

**FIG 4:**
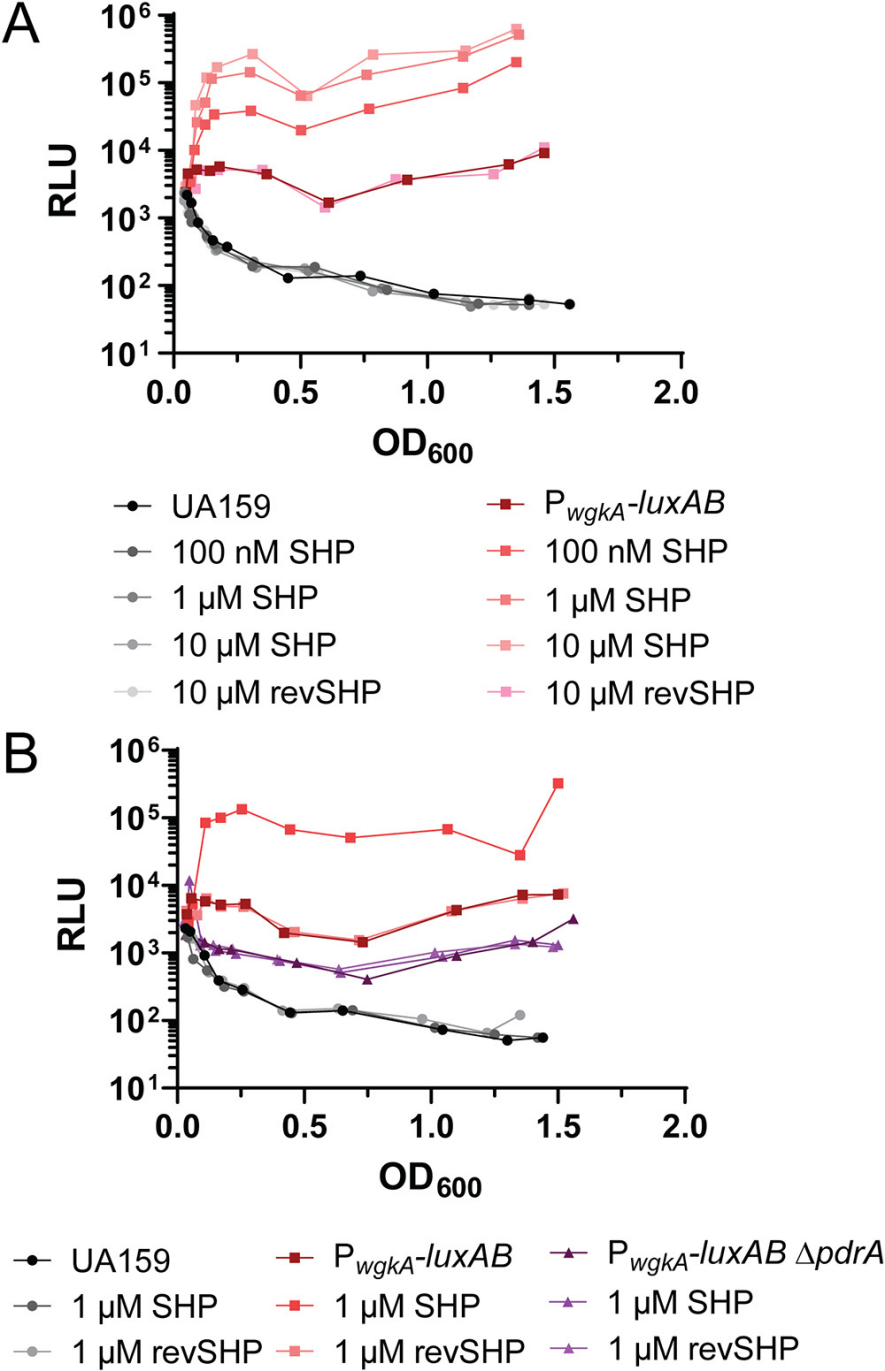
PdrA induces the *wgk* operon upon addition of SHP and is required for induction of the *wgk* operon. (A) P*_wgk_* is induced by the addition of SHP peptide in a dose-dependent manner. Wild-type UA159 and *galK:*:P*_wgkA_-luxAB*-P_c_-*erm* (P*_wgkA_-luxAB*) strains were incubated with increasing concentrations of SHP peptide (no peptide, 100 nM, 1 μM, 10 μM) or 10 μM reverse SHP peptide as a negative control. (B) PdrA is required for induction of the P*_wgkA_* promoter response to SHP. Wild-type UA159, *galK:*:P*_wgkA_-luxAB*-P_c_-*erm* (P*_wgkA_-luxAB*), and *galK:*:P*_wgkA_-luxAB*-P_c_-*erm* Δ*pdrA*::*spec* (P*_wgkA_-luxAB* Δ*pdrA*) strains were incubated without SHP, with 1 μM SHP, or with 1 μM reverse SHP as a negative control. These experiments were performed a minimum of two times with similar results.

To evaluate the regulatory control of *wgk* in response to SHP pheromone and PdrA, a luciferase reporter was constructed that couples the putative promoter of *wgkA* (P*_wgkA_*) to *luxAB* at the *galK* locus of the S. mutans chromosome (*galK*::P*_wgkA_-luxAB*-P_c_-*erm*, BRSM49, Table S1). Cultures of P*_wgkA_-luxAB* were treated with concentrations of SHP or reverse SHP peptide ranging from 0.1 to 10 μM, and relative luciferase activities were measured. Although no induction of P*_wgkA_-luxAB* was observed in response to reverse SHP, cultures displayed a dose-dependent response to SHP, where 10 μM led to a 100-fold induction of luciferase activity and appeared to approach saturation (Fig. 4A). PdrA was required for this response, as a deletion of the regulator negated any response to SHP (Fig. 4B). Together, these data demonstrate that expression of the *wgk* operon relies on PdrA in a SHP-dependent manner.

### Identification of tryglysins, products of *wgk* operons from *S. ferus* and S. mutans.

To identify the mature product of the *wgk* operon, we first focused on *S. ferus*, as the reaction of the RaS enzyme WgkB has been determined from this strain (26). Based on these previous data, a list of possible mature products was assembled computationally. The presence of these candidate peptides in culture supernatants was then assessed using high-performance liquid chromatography (HPLC)-quadrupole time of flight mass spectrometry (Q-TOF-MS). Specifically, *S. ferus* was grown in chemically defined medium (CDM), and aliquots were collected at various growth stages. Each aliquot was both computationally and manually surveyed for HR-MS (high-resolution mass spectrometry) matches to the list of candidate products, which were subsequently targeted for HR-tandem MS (MS/MS) analysis. From these screens, a single parent ion emerged with the accurate *m/z* of 823.3868 and an MS/MS pattern consistent with the C-terminal 7 amino acids (VNSWGKH) of WgkA containing a −4-Da modification (Fig. S3C and Table S2A). The recovery yield of this modified peptide was too low to allow for direct isolation and structural elucidation. We therefore chose to synthetically prepare the putative 7-mer mature product containing the macrocyclic modification and to assess its chromatographic and HR-MS properties in comparison to the authentic material.

**FIG 5:**
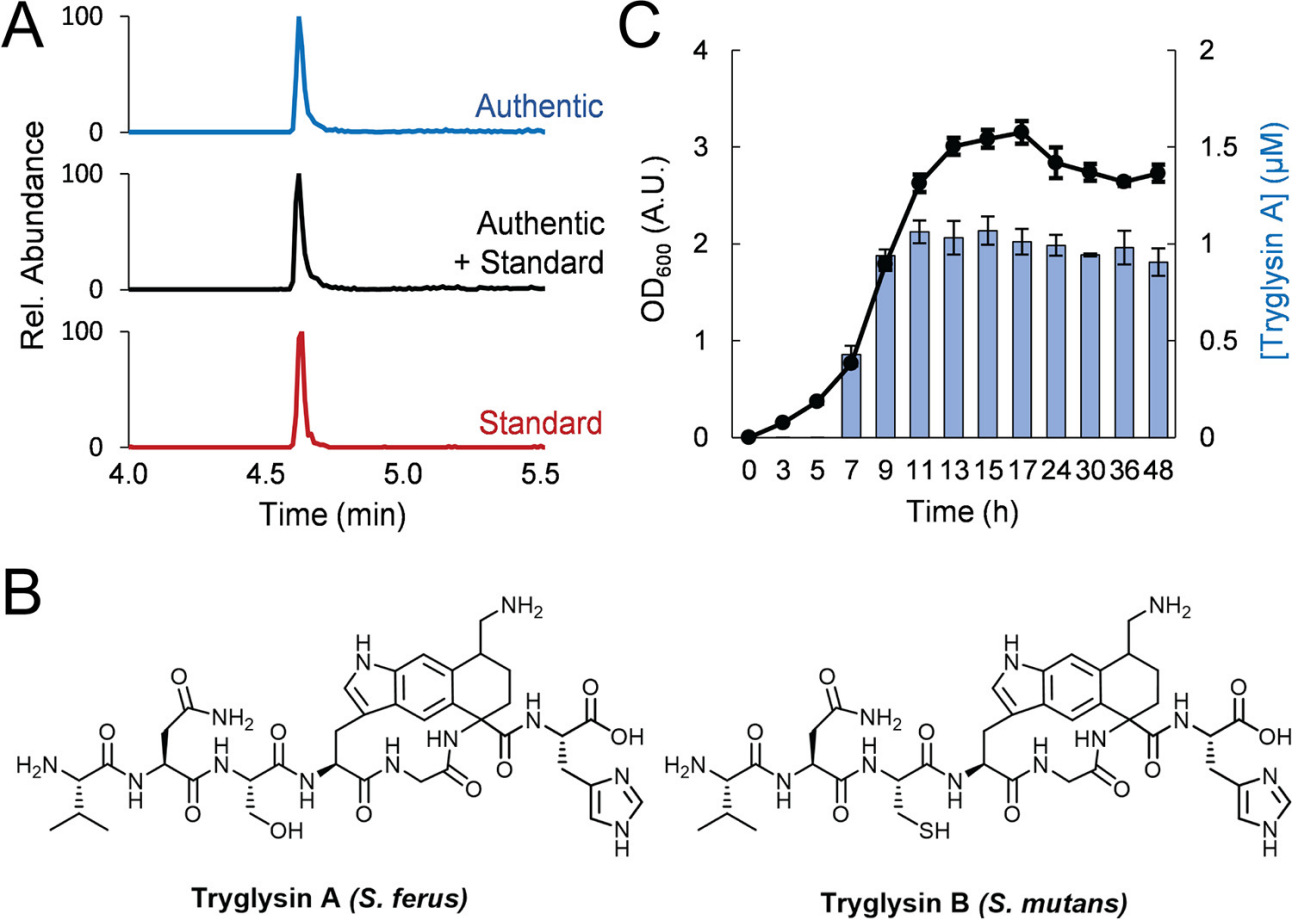
Identification of tryglysin A from *S. ferus*. Shown are elution profiles of synthetic tryglysin A (red trace, standard), authentic tryglysin A isolated from *S. ferus* (blue), and a 1:1 mixture of synthetic and authentic tryglysin A (black), which coelute as a single peak, indicating together with HR-MS and HR-MS/MS data that the two compounds are identical. Rel., relative. (B) Structures of tryglysin A and tryglysin B from *S. ferus* and S. mutans, respectively. The absolute configuration of the α- and δ-carbons of the cross-linked lysine remain to be determined. (C) Quantification of tryglysin A from *S. ferus* (blue bars). OD_600_ is shown as absorbance units (A.U.).

The synthetic 7-mer peptide containing the appropriate modification was prepared using a construct in which *S. ferus wgkA* carrying an N-terminal purification tag, *wgkB*, and *wgkC* were coexpressed from the same plasmid in Escherichia coli. The modified peptide was then isolated using the tag and treated with two different proteases to give the desired 7-mer macrocyclic peptide. HR-MS and tandem HR-MS were consistent with the presence of the tetrahydro[5,6]benzindole modification (Fig. S3D and Table S2B to S2D), which we elucidated previously. This synthetic standard was then compared with the authentic material from *S. ferus*. The two peptides showed identical chromatographic behavior (Fig. 5A); importantly, a 1:1 mixture of synthetic and authentic material coeluted with identical HR-MS and tandem HR-MS parameters (Fig. 5A, Fig. S3C and D, and Table S2A to S2C). Together, these data establish the product of the *S. ferus wgk* cluster as the macrocyclic 7-mer peptide shown (Fig. 5B). We have termed this novel natural product tryglysin A after the tryptophan, glycine, and lysine amino acids that are involved in the macrocycle. Tryglysin A is the founding member of a new class of RiPP natural products, as the macrocyclic modification is unprecedented. The synthetic material also allowed us to quantify tryglysin A production as a function of growth phase. The results show that production commences in early stationary phase, peaking at late exponential phase at ∼1 μM bulk concentration (Fig. 5C). Tryglysin A then persists at fairly high titers during stationary phase.

Repeated efforts to identify a similar product from S. mutans failed. Altering growth medium or temperature or inclusion of SHP during growth were not successful either. At this point, we cannot explain the lack of detection of a similar peptide from S. mutans, especially as the only difference in the C-terminal seven amino acids is the substitution of serine in tryglysin A with cysteine in the corresponding S. mutans peptide. To gain access to the product of the S. mutans *wgk* cluster, we utilized the E. coli heterologous expression system. S. mutans *wgkA*, *wgkB*, and *wgkC* were coexpressed from the same vector in E. coli, and the modified peptide was then isolated as described for *S. ferus*. The resulting 7-mer macrocyclic peptide consists of the sequence VNCWGKH and carries the tetrahydro[5,6]benzindole modification (Table S2B, S2D, and S2E). We have named this peptide tryglysin B as the predicted product of the S. mutans *wgk* cluster (Fig. 5B).

### WGK peptides inhibit growth and increase cell chaining.

**FIG 6:**
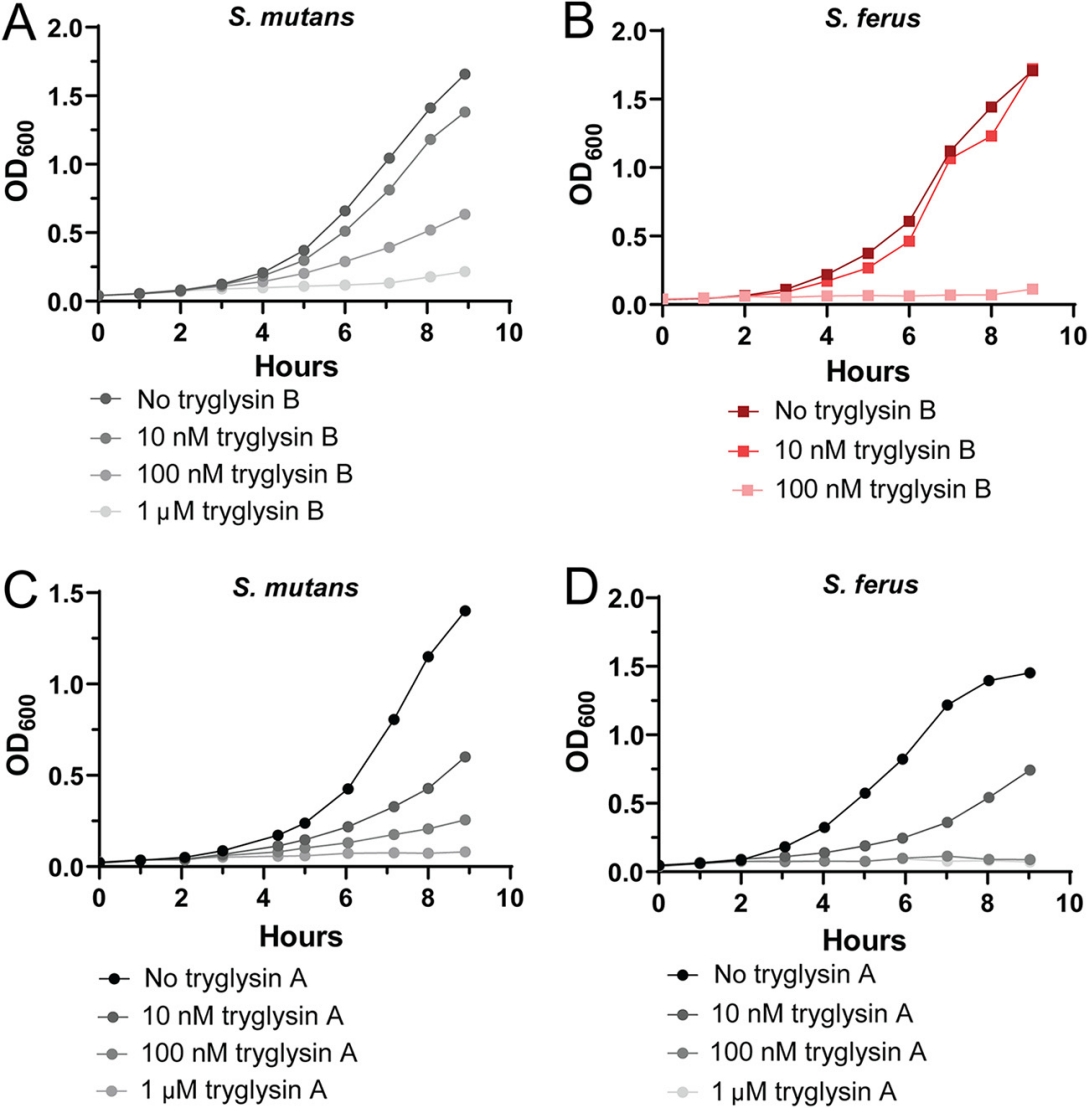
Purified tryglysins inhibit growth of S. mutans and *S. ferus.* Experiments were performed three times with similar results. (A) Tryglysin B inhibits growth of S. mutans UA159 in a dose-dependent manner. Strains were grown in chemically defined media. At 1 h postinoculation from overnight cultures, strains were exposed to the indicated concentrations of tryglysin B. (B) Tryglysin B inhibits growth of *S. ferus* DSM 20646 in a dose-dependent manner. Strains were grown as described above for panel A. (C) Tryglysin A inhibits growth of S. mutans UA159 in a dose-dependent manner. Strains were grown in chemically defined media. At 1 h postinoculation from overnight cultures, strains were exposed to the indicated concentrations of the peptide. (D) Tryglysin A inhibits growth of *S. ferus* DSM 20646 in a dose-dependent manner. Strains were grown as described above for panel C.

**FIG 7:**
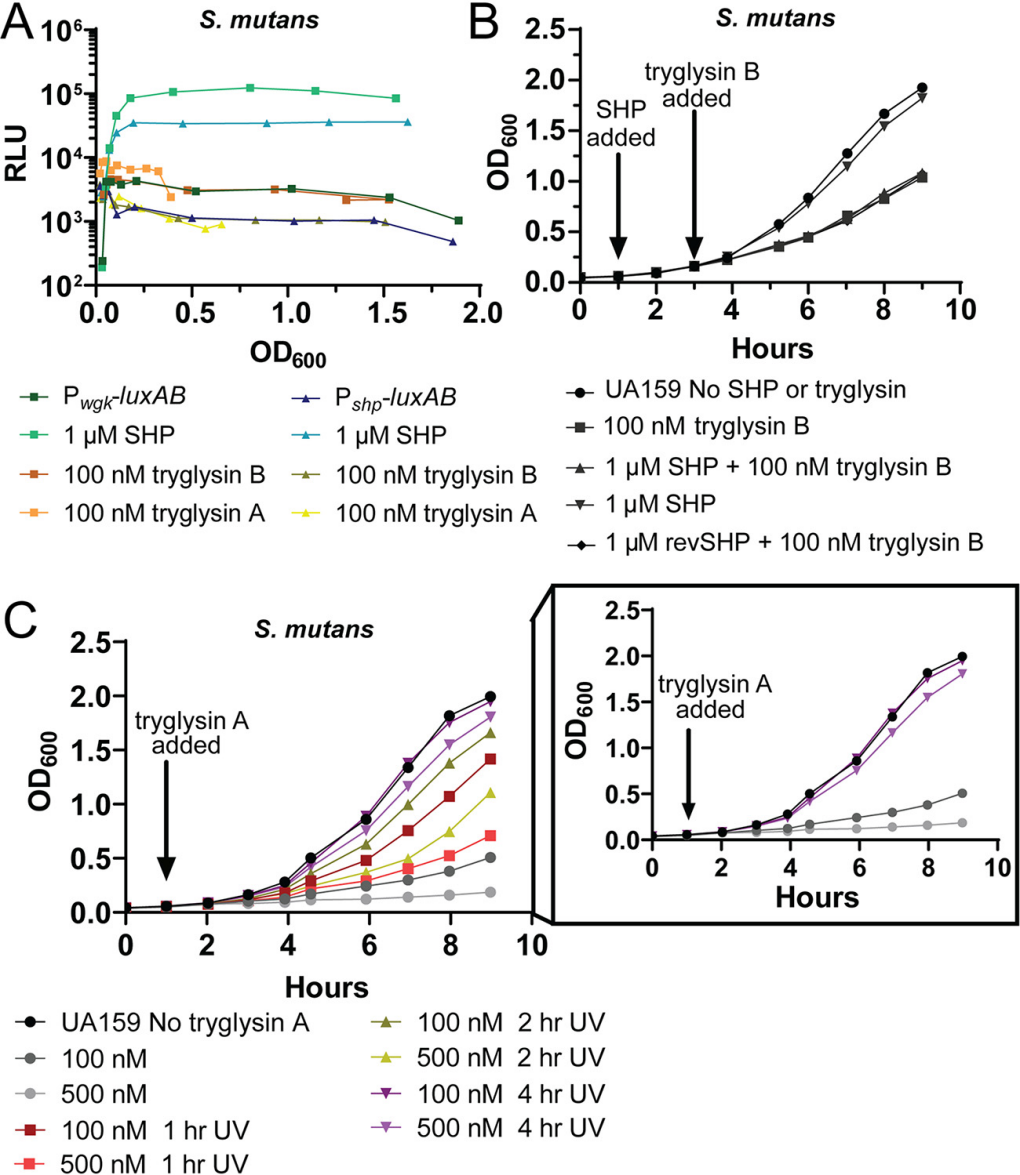
Examination of the PdrA system response to tryglysins. These experiments were performed a minimum of two times with similar results. (A) Tryglysins do not induce the system. Luciferase assay of *galK*::P*_wgkA_-luxAB*-P_c_-*erm* (P*_wgkA_-luxAB*) and *galK*::P*_shp_-luxAB*-P_c_-*erm* (P*_shp_-luxAB*) strains upon addition of 1 μM SHP, 100 nM tryglysin B, or 100 nM tryglysin A or no addition. (B) SHP peptide does not induce immunity to tryglysin B 2 h after SHP induction. S. mutans UA159 was grown for 1 h in chemically defined media after inoculation from overnight cultures. At 1 h, 1 μM SHP or 1 μM reverse SHP (revSHP) peptide was added to the media. After 2 h of induction with peptides, 100 nM tryglysin B was added to the media. (C) Tryglysin A inhibitory activity can be inactivated by exposure to UV light. Growth curve of S. mutans UA159 that was not treated or treated with tryglysin A inactivated for 1 h, 2 h, or 4 h with short-wavelength UV-light. Strain UA159 was treated with 100 or 500 nM tryglysin A. The inset panel is the same growth curve as in panel A, comparing UA159 exposed to untreated tryglysin A or tryglysin A treated with UV light for 4 h.

P*_shp_-luxAB* and P*_wgkA_-luxAB* strains were again employed to test the possibility that tryglysins of S. mutans or *S. ferus* are signals that influence transcription of the regulatory and biosynthetic genes. Although reporters were unresponsive (see Fig. 7A), a clear impact on culture growth was observed upon application of 100 nM tryglysin A or B. Growth inhibition of S. mutans and *S. ferus* cultures was tested with titrations of both tryglysins. At 100 nM either peptide, both S. mutans and *S. ferus* growth was substantially inhibited (Fig. 6), though S. mutans required 1 μM tryglysin B to be completely inhibited over the 9-h growth assay (Fig. 6A).

In the process of purifying tryglysins, it was observed that UV light exposure resulted in degradation of the macrocycle. To ensure that growth inhibition was due to activity of WGK peptides and not a contaminating substance, we took advantage of the sensitivity of the peptides to UV light. Aliquots of tryglysin A were exposed to UV light for time periods up to 4 h and then applied to cultures of S. mutans. Proportionate decrease of inhibitory activity was observed (Fig. 7C), indicating that growth inhibition requires an intact macrocycle.

As antimicrobial biosynthetic genes typically include immunity factors to protect producers from inhibiting their own growth, we tested whether the expression of the *wgk* operon by pretreating with SHP prior to exposure to tryglysin B would protect S. mutans from growth inhibition effects. S. mutans cultures were stimulated with 1 μM SHP for 2 h prior to challenging with 100 nM tryglysin B (Fig. 7B), an induction time that led to maximal transcription of the P*_wgkA_* promoter (Fig. 4A). However, no differences in growth rates were observed after treatment in cultures induced with SHP or reverse SHP (Fig. 7B). From this, we conclude that either S. mutans does not possess an immunity factor to tryglysin B or that the factor was not induced by our experimental set-up.

**FIG 8:**
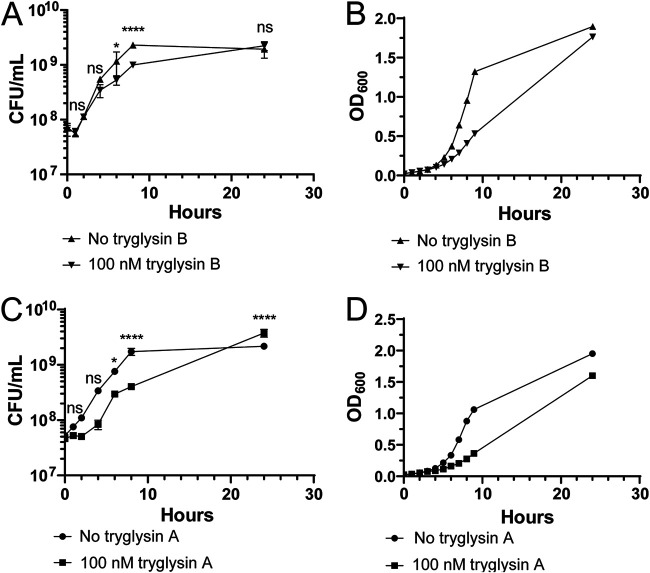
Tryglysins are bacteriostatic for S. mutans. These experiments were performed three times with similar results. Statistical significance was determined via a one-way ANOVA with Sidak’s multiple comparison posttest. (A) CFU/ml assay of S. mutans UA159 in the presence or absence of S. mutans tryglysin B. (B) Parallel growth curve of CFU/ml assay of S. mutans UA159 in the presence or absence of tryglysin B. (C) CFU/ml assay of S. mutans UA159 in the presence or absence of tryglysin A. (D) Parallel growth curve of CFU/ml assay of S. mutans UA159 in the presence or absence of tryglysin A. ns, not significant.

To begin determining a mechanism by which the peptides inhibit growth, S. mutans cultures were treated with 100 nM tryglysin A or B, and viability was ascertained by plating after periods of exposure times. Addition of the peptides resulted in statistically significant lower CFU/milliliter at 6 h and 8 h compared to untreated cultures (Fig. 8A and C), but treated cells were able to overcome this by 24 h (Fig. 8A and 8C). A proportionate inhibition and restoration of growth were observable by optical density measurements of cultures (Fig. 8B and D). These results demonstrate that while tryglysins are inhibitory, eventually S. mutans overcomes this inhibition, whether it be by adaptation of bacterial cells or due to diminished activity of the peptides under the assessed conditions. As cultures continued to grow during treatment, albeit at reduced rates, the overall effects displayed bacteriostatic activities rather than bactericidal.

**FIG 9:**
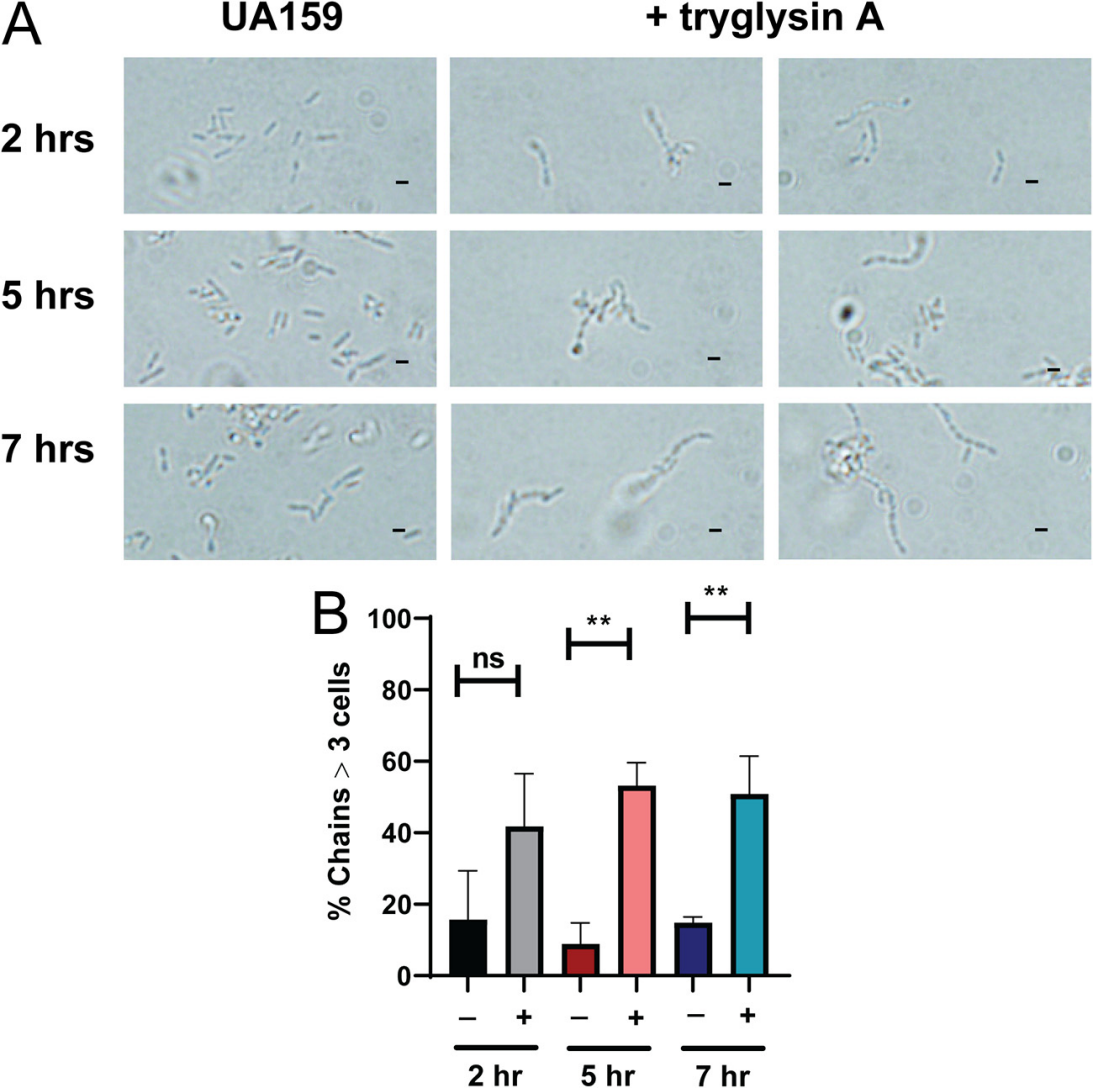
Tryglysins increase cell chaining of S. mutans. Experiments were performed three times with similar results. Microscopy of S. mutans UA159 upon exposure to 100 nM tryglysin A (+ tryglysin A). Fixed cells were observed via a 100× objective at 2 h, 5 h, and 7 h after tryglysin A addition to the media. Brightness and contrast were adjusted for images. Bars, 1 nm. (B) Quantification of chains greater than three cells in length for S. mutans exposed to 100 nM tryglysin A for 2 h, 5 h, or 7 h. A minimum of 100 cells were observed per condition and time point. −, no tryglysin A; +, 100 nM tryglysin A. **, *P* < 0.01; ns, not significant by one-way ANOVA with Dunnet’s multiple comparison posttest.

The impact of tryglysins on gross morphological changes to cell shape was examined at 2, 5, and 7 h after treatment with 100 nM tryglysin A or B by microscopy. Treatment resulted in the appearance of many long chains of cells, correlating with longer treatment times (Fig. 9 and Fig. S6). At 2 h, small chains were observed; this chaining significantly increased upon tryglysin A addition at 5 h and 7 h and appeared to do the same for tryglysin B-treated cells (Fig. 9B and Fig. S6). We conclude that the overall effect on S. mutans morphology is similar with both antibiotic peptides.

10.1128/mBio.02688-20.8 FIG S6 Additional images demonstrating that tryglysins increase cell chaining of S. mutans. Experiments were performed three times with similar results. Live cell microscopy of S. mutans UA159 upon exposure to 100 nM tryglysin B, 100 nM tryglysin A, or no peptide. Cells were observed via a 40× objective at 2 h, 5 h, and 7 h after peptide addition to the media. Scale bar equals 1 nm. Brightness and contrast were adjusted for images. Download FIG S6, PDF file, 0.9 MB. Copyright © 2021 Rued et al. 2021 Rued et al. https://creativecommons.org/licenses/by/4.0/ This content is distributed under the terms of the Creative Commons Attribution 4.0 International license.

### WGK peptides inhibit growth of several streptococcal species, but not E. faecalis or L. lactis.

**FIG 10:**
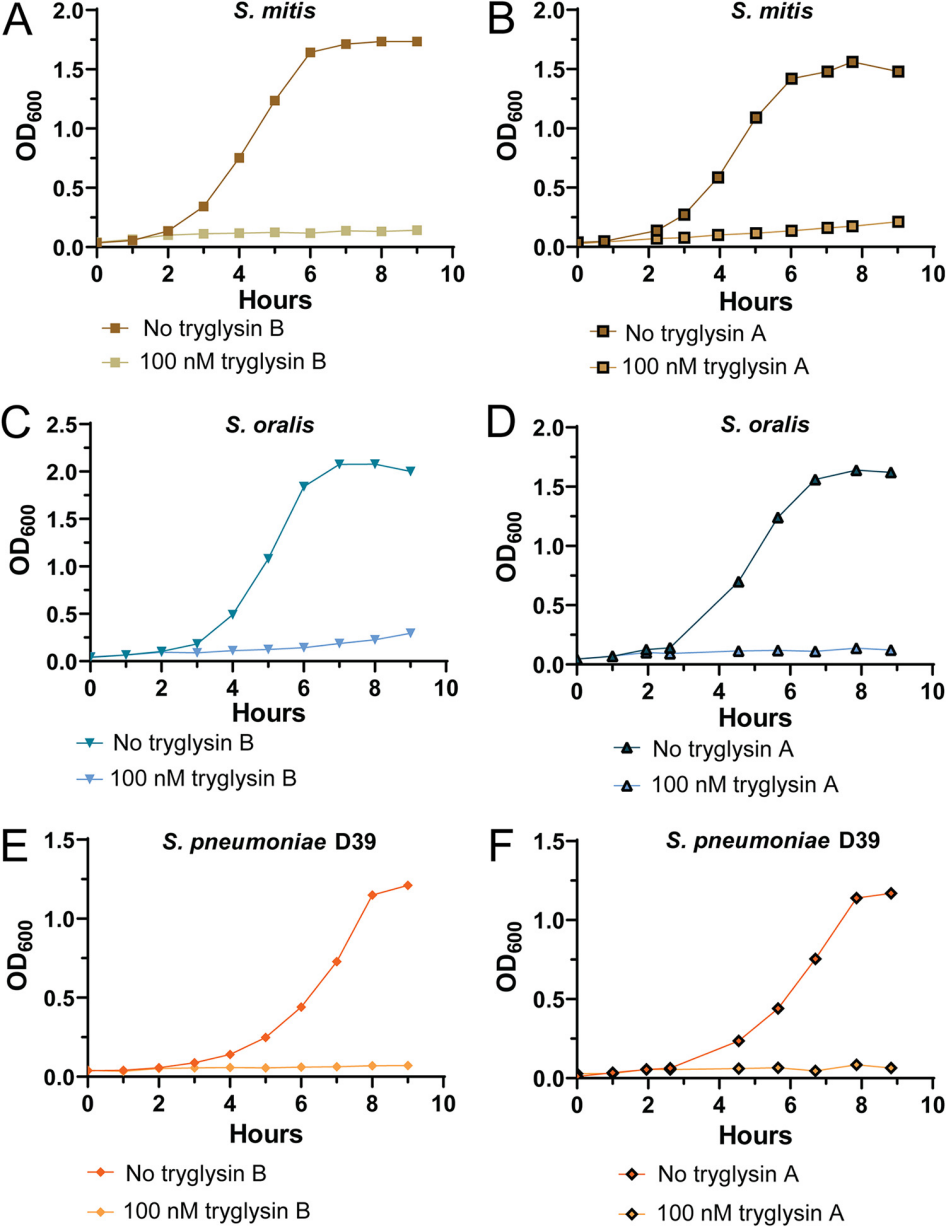
Tryglysins inhibit the growth of S. mitis, S. oralis, and S. pneumoniae D39. Experiments were performed three times with similar results, except for S. pneumoniae, which had two experiments in which inhibition was observed and one experiment in which it was not. For a summary of all tested strains, please see Table 3. (A) Growth curve of S. mitis CCUG 31611 with and without 100 nM tryglysin B. (B) Growth curve of S. mitis CCUG 31611 with and without 100 nM tryglysin A. (C) Growth curve of S. oralis 108 with and without 100 nM tryglysin B. (D) Growth curve of S. oralis 108 with and without 100 nM tryglysin A. (E) Growth curve of S. pneumoniae D39 with and without 100 nM tryglysin B. (F) Growth curve of S. pneumoniae D39 with and without 100 nM tryglysin A.

We extended the evaluation of tryglysin inhibitory activity to other bacterial species. Culture growth rates of several Gram-positive species were evaluated upon treatment with 100 nM each antibiotic. Most susceptible to treatments, with complete growth inhibition at only 100 nM tryglysins, were strains of S. mitis, S. oralis, and S. pneumoniae, each being members of the mitis group (Fig. 10 and Table 3). Tryglysin A displayed greater inhibitory activity against S. agalactiae and to a lower degree, Streptococcus sanguinis and Streptococcus bovis, than variant B at 100 nM (Fig. S5A and B and S5E to H). S. gordonii displayed an extended lag phase in response to both peptides (Fig. S5C and D). Unaffected were cultures of S. pyogenes, Lactococcus lactis, and Enterococcus faecalis (Fig. S4A to F). Thus, for the broad panel of lactic acid bacteria tested, tryglysins displayed a range of specificity for inhibiting streptococcal species but were inert toward E. faecalis and L. lactis.

**TABLE 3:** Bacterial species tested for inhibition by tryglysins A and B^*a*^

Species tested	Inhibition by tryglysin A	Inhibition by tryglysin B
E. faecalis V583	−	−
L. lactis ATCC 11454	−	−
S. pyogenes NZ131	−	−
S. ferus DSM 20646	**++**	**++**
S. mutans UA159	**++**	**++**
S. sanguinis ATCC 10556	**++**	**++**
S. gordonii Challis Bt	**+**	**+**
S. mitis CCUG 31611	**++**	**++**
S. oralis 108	**++**	**++**
S. pneumoniae D39	**++**^*b*^	**++**
S. agalactiae A909	**++**	**+**
S. bovis JB1	**+**	**+**

a Summary of data shown in Fig. 6 and 10 and Fig. S4 and S5. Level of inhibition upon inoculation with 100 nM tryglysin A or B. −, not inhibited; +, mildly inhibited; ++, inhibited.

b For S. pneumoniae D39, two experiments showed inhibition with 100 nM tryglysin A, one experiment did not.

10.1128/mBio.02688-20.6 FIG S4 S. mutans and *S. ferus* tryglysins do not inhibit the growth of E. faecalis, L. lactis, or S. pyogenes. Experiments were performed three times with similar results. For a summary of all tested strains, please see Table 3. (A) Growth curve of E. faecalis V583 with and without 100 nM tryglysin B. (B) Growth curve of E. faecalis V583 with and without 100 nM tryglysin A. (C) Growth curve of L. lactis ATCC 11454 with and without 100 nM tryglysin B. (D) Growth curve of L. lactis ATCC 11454 with and without 100 nM tryglysin A. (E) Growth curve of S. pyogenes NZ131 with and without 100 nM tryglysin B. (F) Growth curve of S. pyogenes NZ131 with and without 100 nM tryglysin A. Download FIG S4, PDF file, 0.3 MB. Copyright © 2021 Rued et al. 2021 Rued et al. https://creativecommons.org/licenses/by/4.0/ This content is distributed under the terms of the Creative Commons Attribution 4.0 International license.

10.1128/mBio.02688-20.7 FIG S5 Tryglysins display various effects on the growth of S. sanguinis, S. gordonii, S. agalactiae, and S. bovis. Experiments were performed three times with similar results. For a summary of all tested strains, please see Table 3. (A) Growth curve of S. sanguinis ATCC 10556 with and without 100 nM tryglysin B. (B) Growth curve of S. sanguinis ATCC 10556 with and without 100 nM tryglysin A. (C) Growth curve of S. gordonii Challis Bt with and without 100 nM tryglysin B. (D) Growth curve of S. gordonii Challis Bt with and without 100 nM tryglysin A. (E) Growth curve of S. agalactiae A909 with and without 100 nM tryglysin B. (F) Growth curve of S. agalactiae A909 with and without 100 nM tryglysin A. (G) Growth curve of S. bovis JB1 with and without 100 nM tryglysin B. (H) Growth curve of S. bovis JB1 with and without 100 nM tryglysin A. Download FIG S5, PDF file, 0.3 MB. Copyright © 2021 Rued et al. 2021 Rued et al. https://creativecommons.org/licenses/by/4.0/ This content is distributed under the terms of the Creative Commons Attribution 4.0 International license.

## DISCUSSION

**FIG 11:**
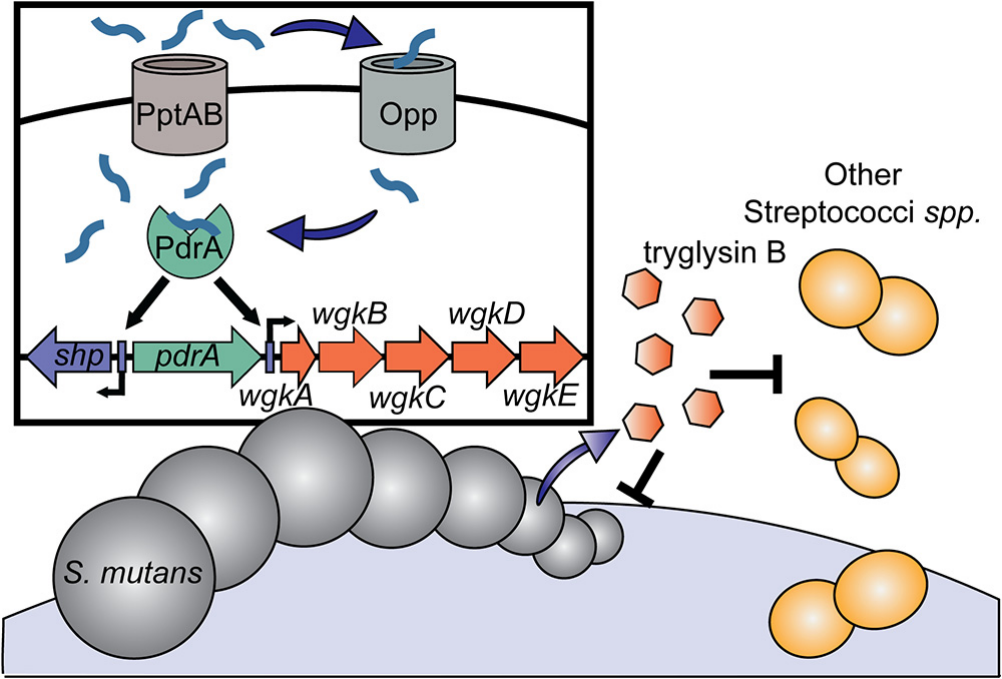
Model of the PdrA/WGK system. The inset shows the PdrA signaling system. Upon receipt of an unknown signal, SHP production is induced in S. mutans. SHP is exported by the PptAB exporter and reimported or imported into nearby cells via the Opp transporter. SHP binds to PdrA, and PdrA activates transcription of the *shp* and the *wgk* operon. The *wgk* operon directs the production of mature tryglysin B, which is exported out of the cell. Tryglysin B then inhibits growth of neighboring competing streptococci or performs a yet unidentified function.

Although genetic linkage between Rgg and RaS/RiPP systems has become evident in recent years (26, 31, 32), only a few studies have addressed whether SHP/Rgg systems directly regulate production of RaS-RiPP operons (10, 25). Here, we demonstrate that the PdrA system directly regulates the *wgk* RaS-RiPP operon in S. mutans UA159. Like other Rgg QS systems in streptococci, S. mutans UA159 produces an SHP encoded by a short open reading frame next to PdrA (Fig. 1), and addition of this short peptide to reporter strains resulted in PdrA-dependent upregulation of promoter targets and autoinduction of SHP (Fig. 2 and 4) (9, 11, 12, 14, 15). Also, similar to previously described SHP/Rgg systems, the PptAB and Opp transporters are required for the import and export of the SHP peptide, respectively (see Fig. S2 in the supplemental material) (9, 11, 12, 50). Induction of this operon presumably results in the production of the S. mutans tryglysin, although detection of this peptide in CDM supernatants has eluded us so far. It may be that the tryglysin is degraded in CDM by a target enzyme, is produced maximally at certain times after SHP induction, is not stable in our detection assays, or is simply produced in too low of an amount to detect above background peptides in CDM. These possibilities are not necessarily mutually exclusive and will take further investigation to disentangle. Nevertheless, we have demonstrated that the PdrA system in S. mutans directly regulates the WGK RaS-RiPP system and constitutes an SHP/Rgg system like those in other streptococci. A model of this system is presented in Fig. 11.

A leading aspect of this study is the physiological consequence of induction of the *wgk* RaS-RiPP system. Genes involved in competence, adhesion, and metabolism were found to be differentially regulated upon stimulation with SHP. Competence gene regulation typically coincides with production of antimicrobial agents leading to lysis of neighboring cells and subsequent acquisition of released DNA. PdrA does not appear to activate transformation in a way seen for that of the ComR/XIP quorum-sensing systems (another Rgg-like system). We wonder whether the inversed expression patterns of *wgk* and competence versus adherence indicate a decision branchpoint of distinct lifestyles. Antimicrobial production may be more effective, or possibly less harmful to the producing bacteria, if self-aggregation or adhesion to surfaces is avoided. Further studies are needed concerning these aspects, particularly how posttranscriptional expression of *irvA* and *gbpC* are impacted by PdrA and *wgk*.

We found that addition of pure tryglysins to S. mutans and other streptococcal strains inhibits growth of these organisms (Fig. 6 and 10, Fig. S5, and Table 3). This activity is reminiscent of mutacins (35, 60), and involvement of an Rgg regulator for tryglysin induction is similar to the linkage of MutR to mutacin I, II, and III (21, 61). However, tryglysins have properties that are distinct from previously identified mutacins. First, although they make use of a RiPP operon for their synthesis, like lantibiotics, they do not possess the characteristic lanthionine and methyllanthionine residues of these bacteriocins (60, 62) (Fig. 5B). Instead they harbor an unprecedented tetrahydro-[5,6]benzindole motif, which qualifies tryglysin A and B as the founding members of a new subclass of RiPPs. Second, the *wgk* locus appears to be conserved in S. mutans, as previous analysis and nucleotide BLAST indicates high levels of conservation of the *wgk* operon in S. mutans strains (26). This is distinct from mutacins, which vary in their conservation and can be strain specific (60).Third, and paradoxically, the tryglysins display inhibitory activity toward their producing strains (Fig. 6). Typically, streptococcal antimicrobial biosynthetic gene clusters include immunity genes that allow producer strains to be resistant to the natively produced mutacins (60). We originally theorized that induction of the PdrA system would lead to production of an immunity factor by S. mutans UA159, but in initial experiments we did not observe any SHP-induced immunity to tryglysin B (Fig. 7B). This does not rule out that an immunity factor might still exist for these peptides and that we have not identified the correct conditions to observe immune activity. Ultimately, collateral damage of self-inhibition may still be beneficial to the producer if competitors are more susceptible. We did observe that S. mutans was able to overcome this inhibition to reach similar levels to untreated cells by 24 h of growth (Fig. 8). We attempted multiple experiments to observe tryglysin activity *in vivo* from *S. ferus* or S. mutans via deferred antagonism assays or application of supernatants to test strains. However, we have not observed consistent inhibition *in vivo* that we can attribute to tryglysin production and believe that we have yet to determine the correct conditions for observing this inhibition from live S. mutans or *S. ferus* cells. These observations raise the question of whether tryglysins are meant simply to slow the growth of competitor strains or if they have a more complex function (Fig. 11). A final alternative that needs to be investigated is whether tryglysins also represent fratricidal agents (34, 63). The options above are not mutually exclusive, and it is possible that tryglysins serve multiple functions. To answer these questions, further experiments investigating how these peptides alter a complex oral community predominated by streptococci, such as in the *in vitro* system developed by Edlund et al. are warranted (64).

We also observed that tryglysins strongly inhibited members of the mitis group, such as S. mitis, S. oralis, and S. pneumoniae (Fig. 10). S. sanguinis was also inhibited, although this strain did not grow optimally in our chemically defined medium (Fig. S5A and B). These organisms colonize the oral microbiome earlier than S. mutans and can inhibit S. mutans growth via hydrogen peroxide production (39, 65, 66). As such, inhibiting the growth of these competitor strains could be advantageous to S. mutans (66, 67).

Another aspect of interest in this study is the observation that tryglysin treatment results in increased cell chaining in S. mutans (Fig. 9). We do not know the underlying mechanism, but it is reminiscent of morphological changes in streptococci that occur upon disruption of cell division or stress induction (68, 69). To understand what the impact of the addition of these peptides are, RNA-seq could be performed on strains exposed to inhibitory concentrations of tryglysins. This would uncover genes induced upon addition of these peptides and might provide clues toward the mechanisms that underlie the observed morphological changes.

One caveat to these observations is the fact that we have not directly observed inhibition of other species upon induction of the PdrA system. We have observed that *in vitro*, SHP addition to S. mutans cultures increases the doubling time relative to strains without SHP (Fig. S1B), but not to the extent of the inhibition we observed with purified tryglysins. As previously mentioned, attempts to examine tryglysin activity *in vivo* have been unsuccessful. These data suggest that tryglysins are likely produced by S. mutans under our *in vitro* conditions but that the level is somehow regulated by S. mutans to balance *wgk* induction with growth. Perhaps there is a turnover system of tryglysin induced by S. mutans, which regulates the native level of these peptides. This would not be unprecedented, as other small peptide systems have been found to have associated turnover enzymes (70, 71). Further experiments concerning a possible turnover of tryglysins are current goals we are pursuing.

In short, this study reports the discovery and first demonstration of the physiological relevance of tryglysins from S. mutans and *S. ferus*. We found that the PdrA system in S. mutans behaves similarly to other streptococcal Rgg systems and that the *wgk* biosynthetic operon is linked to induction of this system. We have also found that the tryglysins formed by this biosynthetic operon inhibit the growth of a panel of streptococcal species when added *in vitro*, which has implications for the rest of the previously identified RaS-RiPP clusters and the molecules they produce. Tryglysins represent a new class of antimicrobial RiPPs, distinct from previously characterized classes produced by RiPP operons (72). Further investigation of these systems in other streptococci is ongoing. It will be exciting to see the physiological impact of other Rgg-linked RaS-RiPP systems in streptococci and whether the impact they have on other bacterial species is similar to that of tryglysins. Future studies will determine how these peptides contribute to streptococcal physiology and their importance in polymicrobial communities.

## MATERIALS AND METHODS

### Bacterial strains, plasmids, and growth conditions.

Bacterial strains and plasmids used in this study are listed in Table S1 in the supplemental material. S. mutans strains were derived from S. mutans UA159 (73). S. mutans, L. lactis, E. faecalis, and other streptococcal strains listed in Table S1 were grown on Todd-Hewitt (TH) plates (BD Biosciences) with 1.4% Bacto agar (BD Biosciences) and 0.2% yeast (BD Biosciences), Todd-Hewitt broth with 0.2% yeast (THY), or in chemically defined medium (CDM) plus 1% glucose at 37°C in an atmosphere of 5% CO_2_. The components and recipe for CDM used were as described previously (9). E. coli strains for plasmid construction and propagation were derived from E. coli BH10c or DH5α (74). E. coli strains were grown on Luria-Bertani (BD Biosciences) plates with 1.4% Bacto agar at 37°C or in Luria-Bertani (LB) broth or terrific broth with shaking at 200 rpm. When required, spectinomycin (100 μg/ml), erythromycin (0.5 μg/ml for S. mutans; 500 μg/ml for E. coli), kanamycin (50 to 100 μg/ml), and chloramphenicol (3 μg/ml) were added to S. mutans or E. coli culture media. Ampicllin (100 μg/ml) was added to E. coli cultures when required.

### Construction of S. mutans strains.

Transformation of S. mutans UA159 to obtain derivative strains (Table S1) was performed with linear DNA amplicons or plasmids as described previously (75), with the exception of strain GS105 which was obtained via electroporation (described later). For strains BRSM05 (Δ*pdrA*::*spec*) and BRSM08 (Δ*wgkAB*_N-term_::*spec*), linear DNA amplicons were constructed as follows. Fragments from UA159 genomic DNA and pL12Spec were amplified using primers in Table S1. Resulting fragments were digested using restriction enzymes listed in Table S1, and fragments were subsequently ligated together using T4 DNA ligase (New England Biolabs [NEB]) according to the manufacturer’s instructions. For strain BRSM08, we noted that although we constructed primers to replace only the Δ*wgkA* coding sequence with a spectinomycin cassette, transformation of this construct resulted in replacement of the coding sequence of *wgkA* and the first 992 nucleotides of *wgkB* with the spectinomycin cassette. More specifically, the spectinomycin cassette from pLZ12spec was fused to the following intergenic sequence (5′ to 3′) TAAAAAAATTGAAAAAATGGTGGAAACACTTTTTTAATTTTTTTGTTTTATTATTTAATATTTGGGAAATATTCATTCTAATTGGTAATCAGATTTTAGAAAACAATAAACCCTTGCGTCGACG, which was fused to the *wgkB* coding sequence starting at 993 bp. This was determined by Sanger sequencing of the construct and surrounding regions. For strains BRSM38, BRSM49, and BRSM69, linear DNA amplicons were constructed via PCR amplification of DNA fragments using primers in Table S1, subsequent PCR purification with a DNA Clean & Concentrator kit (Zymo Research) and then Gibson assembly using NEBuilder HiFi DNA Assembly MasterMix (NEB) according to manufacturer’s instructions. For strain JCC298, Δ*oppD*::cat was constructed as follows. Fragments from UA159 genomic DNA and from pEVP3 were amplified using primers in Table S1. Resulting fragments were digested using restriction enzymes listed in Table S1, and fragments were subsequently ligated together using T4 DNA ligase according to the manufacturer’s instructions. To obtain strain GS105, strain JCC298 was electroporated with pJC301 and pGS101 using a Bio-Rad Gene Pulser II electroporation system (Bio-Rad) according to the manufacturer’s instructions. Constructed strains were confirmed by PCR and/or Sanger sequencing corresponding to the linear amplicon and surrounding regions.

### Construction of plasmids pGS101, pGS103, pJC300, and pJC301 in E. coli.

The following plasmids were obtained via electroporation of the final construct into E. coli BH10c using a Bio-Rad Gene Pulser II electroporation system. Constructed plasmids were confirmed by PCR and/or Sanger sequencing. Plasmids were constructed as follows.

To obtain pGS101, pWAR303 was propagated in E. coli BH10c cells and extracted using a GeneJET Plasmid MiniPrep kit (Thermo-Fisher) according to manufacturer’s instructions. The P*_wgk_* promoter was amplified from UA159 genomic DNA by PCR with primers GS203/GS204. The resulting pWAR303 plasmid and P*_wgk_* promoter were digested with PstI and SalI and purified with the DNA Clean & Concentrator kit (Zymo Research), and the fragments were ligated together using T4 DNA ligase (NEB). To obtain pGS103, pWAR303 was obtained as previously mentioned. The P*_shp_* promoter was amplified from S. mutans UA159 genomic DNA by PCR with primers GS206/GS207. pWAR303 and P*_shp_* promoter were digested with PstI and SalI and purified, and fragments were ligated together with T4 DNA ligase. To obtain pJC300, pLZ12Spec plasmid was propagated in BH10c cells and extracted as previously described. The P*_syncat_*-cat cassette was obtained from pEVP3 by PCR using primers JC208/JC412. pLZ12Spec and P*_syncat_*-cat cassette were digested using EcoRI, purified, and ligated together. To obtain pJC301, pJC300 was PCR amplified using primers JC413/JC414. The resulting fragment was purified and ligated together using T4 DNA ligase.

### Construction of pRSFDuet-1_6HMBP, pRSFDuet-1_6HMBP*wgkA*_*wgkBC*_DSM20646, and pRSFDuet-1_6HMBP*wgkA*_*wgkB*-rbs-*C*_UA159.

The following plasmids were obtained via transformation of the final construct into chemically competent E. coli DH5α cells via heat shock. All PCR products were purified using the Qiagen PCR purification kit (Fisher Scientific). The Qiagen Gel Extraction kit (Thermo-Fisher) was used to purify DNA fragments and plasmids following digestion with restriction enzymes. Prior to ligation or Gibson Assembly, all linearized vectors were treated with recombinant shrimp alkaline phosphatase (rSAP) (NEB). All ligation reactions were performed with T4 DNA Ligase (NEB), and all DNA assemblies were performed with Gibson Assembly Master Mix (NEB). All cloning reagents were used according to instructions. Assembled plasmids were confirmed by Sanger sequencing. Plasmids were constructed as follows.

To obtain pRSFDuet-1_6HMBP, pBAD6HMBPP_HISTFLAG was purified using a Qiagen Gel Extraction kit (Thermo-Fisher) according to the manufacturer’s instructions and digested with restriction enzymes NcoI and BamHI (NEB) to yield a DNA fragment encoding a 6×His-MBP tag containing a polylinker and HRV-3C protease cleavage site. The fragment was then inserted by ligation into NcoI/BamHI-digested pRSFDuet-1.

To obtain pRSFDuet-1_6HMBP*wgkA*_*wgkBC*_DSM20646, a DNA fragment containing *wgkA* and appropriate overlap regions was PCR amplified with primers *wgkA*_DSM20646_F/*wgkA*_DSM20646_R from Streptococcus ferus DSM20646 genomic DNA that was isolated using the Wizard Genomic purification kit (Promega). A *wgkBC* DNA fragment was generated in the same way, but with primers *wgkBC*_DSM20646_F/*wgkBC*_DSM20646_R. First, vector pRSFDuet-1_6HMBP was linearized with restriction enzymes BamHI and PstI. The digestion product was then combined with the *wgkA* PCR fragment by Gibson Assembly, attaching *wgkA* to the 6HMBP coding sequence in MCS1. The *wgkA*-containing vector was subsequently treated with NdeI and XhoI to open up MCS2 into which the *wgkBC* fragment was inserted by Gibson Assembly.

To construct pRSFDuet-1_6HMBP*wgkA*_*wgkB*-rbs-*C*_UA159, the *wgkA*, *wgkB* and *wgkC* genes were obtained as synthetic DNA fragments (GENEWIZ) and codon optimized for expression in E. coli. The *wgkA* gene was PCR amplified from its synthetic DNA fragment using primers *wgkA*_DSM20646_F/*wgkA*_DSM20646_R, followed by digestion with BamHI and PstI. The insert was introduced into a BamHI/PstI-digested pRSFDuet-1_6HMBP vector by ligation. A DNA fragment with a ribosomal binding site (rbs) sequence inserted between genes *wgkB* and *wgkC* was constructed by overlap extension PCR. The *wgkB* portion was PCR amplified from the synthetic *wgkB* fragment using primers *wgkB*_UA159_F/*wgkB*-rbs_UA159, which appended an overlap region with a rbs to its 3′ end. The *wgkC* portion featuring a complementary rbs-containing sequence at its 5′ end was PCR amplified from the synthetic *wgkC* fragment using primers rbs-*wgkC*_UA159/*wgkC*_UA159_R. The two fragments were then fused in a final PCR using primers *wgkB*_UA159_F/*wgkC*_UA159_R, generating the single *wgkB*-rbs-C fragment. This PCR product was subsequently digested with NdeI and XhoI as was the *wgkA*-containing pRSFDuet-1_6HMBP vector. The insert was ligated with vector to yield the final plasmid.

### Synthesis of SHP, revSHP, and XIP peptides.

Synthetic peptides were purchased from ABClonal (Woburn, MA) or NeoScientific (Cambridge, MA). Purities and preparations used in assays were greater than 70%. SHP and reverse SHP (revSHP) peptides were reconstituted as 7 mM stock in dimethyl sulfoxide (DMSO) and stored in aliquots at −20°C. Subsequent dilutions for working stocks (100 μM) were made in DMSO and stored at −20°C. The UA159 XIP peptide was reconstituted as a 1 mM stock in DMSO and stored in aliquots at −20°C. The sequences of synthetic peptides are listed in Table 1.

### Bioinformatics analysis of *shp*, *wgkA*, and PdrA in S. mutans UA159.

The coding sequence for the cognate *shp* of PdrA (SMU_1509) has been previously documented (45). The *wgkA* coding sequence was identified based on previously published work on WGK from *S. ferus* (26). Promoter prediction of *shp* and *wgkA* was performed by input of 150 bp upstream of the *shp* and *wgkA* ATG site into the BPROM online server as described previously. The predicted PdrA consensus sequence for binding was identified based on nucleotide alignment of the identified *shp* and *wgkA* promoters via the Clustal Omega webserver with default parameters for amino acid alignment (https://www.ebi.ac.uk/Tools/msa/clustalo/) (46), and previous observation that Rgg transcriptional regulators bind upstream of their regulated promoters near the −35 site (76). The homology of PdrA to previously identified regulators in the S. pyogenes strain NZ131 was determined via blastp on the NCBI webserver (https://blast.ncbi.nlm.nih.gov/Blast.cgi?PAGE=Proteins) of the SMU_1509 amino acid sequence (accession number WP_002352338.1) with Streptococcus pyogenes NZ131 (taxid:471876) chosen as the “search set” for the search. All other parameters in blastp were set to default (77). To determine the conservation of the *pdrA* and *wgk* locus in streptococci, the UA159 reference genome (NC_004350.2) at the positions 1430604 to 1435076 (corresponding to the nucleotide sequence of *shp*, *pdrA*, and the *wgk* operon) were analyzed by input of the sequence into nucleotide BLAST, specifying the organism as “Streptococcus (taxid:1301).” Other parameters for BLASTN were set to default (78). To determine conservation of the putative PdrA binding site, 83 bp upstream of the *wgk* ATG start site was taken (corresponding to positions 1434152 to 1434070 in the UA159 reference genome) and analyzed by input into BLAST, specifying the organism as “Streptococcus (taxid:1301).” The resulting alignments were examined for conservation of the AATTGT**X**TATATGGGAT sequence. Other BLASTN parameters were set to default (78).

### General mass spectrometry and purification procedures.

HPLC-coupled high-resolution (HR) MS and tandem HR-MS were carried out on an Agilent 6540 accurate-mass quadrupole time of flight (Q-TOF) MS instrument (Agilent), consisting of an automated liquid sampler, a 1260 Infinity Series LC system, a diode array detector, a JetStream electron spray ionization (ESI) source, and the 6540 Series Q-TOF.

HPLC purifications were carried out on an Agilent 1260 Infinity Series analytical or preparative HPLC system (Agilent) equipped with a temperature-controlled column compartment, a diode array detector, and an automated fraction collector. The analytical system was also equipped with an automated liquid sampler.

### Analytical-scale tests to confirm activity of WgkBC in E. coli.

To confirm that WgkBC from *S. ferus* or S. mutans is active in the context of the E. coli cell, heterologous coexpression was first tested on a small scale. The DSM20656 or UA159 coexpression vector, along with pDB1282 (*isc* operon), was transformed into E. coli BL21(DE3) by heat shock and plated onto LB agar supplemented with 100 μg/ml ampicillin (Amp) and 50 μg/ml kanamycin (Kan). A 15-ml culture tube containing 5 ml LB (plus 50 μg/ml Kan and 100 μg/ml Amp) was inoculated with a single colony and grown overnight at 37°C with shaking. The following morning, 0.5 ml of overnight culture was used to inoculate a 125-ml flask containing 50 ml terrific broth (TB, plus Kan/Amp). This 50-ml culture was grown at 37°C with shaking to an optical density at 600 nm (OD_600_) ≈ 0.4, at which point it was supplemented with 0.05 mM FeCl_3_ and 0.05% arabinose to induce expression of the *isc* operon. The culture was then grown to an OD_600_ ≈ 0.8 at which point it was supplemented with 0.5 mM isopropyl-β-d-thiogalactopyranoside (IPTG) to induce expression of the 6HMBP*wgkA*, *wgkB*, and *wgkC* genes. Growth was continued at 37°C for 18 h. Cells were harvested by centrifugation (30,000 × *g*, 30 min, 4°C). A typical yield was 100 to 200 mg cell paste per 50-ml culture.

Cell paste was resuspended in lysis buffer (1 ml/200 mg cell paste), which consisted of 50 mM Na_2_PO_4_, 300 mM NaCl, 10 mM imidazole (pH 8) (NPi-10) containing 10% (vol/vol) BugBuster 10× protein extraction reagent (Millipore-Sigma). Benzonase-Nuclease (25 U/μl) and protease inhibitor cocktail (Sigma) were added at 1 μl/ml and 10 μl/ml, respectively. The suspension was incubated on ice on a rocking platform for 30 min. Crude cell lysate was centrifuged to remove cell debris. Clarified lysate was loaded onto a HisPur Ni-NTA (nickel-nitrilotriacetic acid) spin column (0.2-ml resin bed volume, Thermo-Fisher), which had been equilibrated with NPi-10. Spin purification was carried out as per manufacturer’s instructions. Briefly, once loaded, the resin was washed with 5 resin bed volumes of NPi-10, followed by 5 volumes of wash buffer consisting of 50 mM Na_2_PO_4_, 300 mM NaCl, 20 mM imidazole (pH 8) (NPi-20). 6HMBPWgkA was eluted from the resin using 2 to 4 volumes of elution buffer consisting of 50 mM Na_2_PO_4_, 300 mM NaCl, 500 mM imidazole (pH 8) (NPi-500). Centrifugation for all steps was carried out at 700 × *g* for 2 min. Protein elution was monitored by Bradford Reagent (Thermo-Fisher). Isolated 6HMBPWgkA was exchanged into cleavage buffer (50 mM Tris-HCl, 150 mM NaCl [pH 8]) using a PD-10 desalting column (GE Healthcare) following manufacturer’s instructions.

Buffer-exchanged 6HMBPWgkA fusion protein was treated with HRV-3C protease to remove the 6HMBP tag. The HRV-3C-cleaved WgkA peptide was subjected to HPLC−Q-TOF-MS analysis. Samples were injected onto a Phenomenex Jupiter C_18_ 300-Å (5-μm, 4.6 × 150 mm) column operating at 0.6 ml/min with H_2_O and methyl cyanide (MeCN) (both containing 0.1% formic acid [FA]) as mobile phases. The elution program consisted of 8% MeCN for 8 min, 8 to 60% MeCN over 12 min, 60 to 100% MeCN over 4 min, and finally 100% MeCN for 6 min.

### Preparation of synthetic tryglysin A.

The E. coli BL21(DE3) strain carrying pRSFDuet-1_6HMBP*wgkA*_*wgkBC*_DSM20646 and pDB1282 was streaked out from glycerol stock onto an LB agar plate supplemented with Kan (50 μg/ml) and Amp (100 μg/ml) and incubated at 37°C overnight. A single colony was then used to inoculate a 250-ml flask containing 100 ml LB (plus 50 μg/ml Kan and 100 μg/ml Amp). This seed culture was then used to inoculate 1.6 liter TB in a 4-liter flask or 0.8 liter TB in a 2-liter flask at 1% dilution. Expression cultures were grown continuously at 37°C with shaking. Arabinose and FeCl_3_ were added to the culture at an OD_600_ ≈ 0.4 at final concentrations of 0.05% and 0.05 mM, respectively. IPTG was added to the culture at an OD_600_ ≈ 0.8 at a final concentration of 0.5 mM. Following 18 h of growth after induction with IPTG, cells were harvested by centrifugation (15,000 × *g*, 30 min, 4°C) and frozen at −80°C. A typical yield was 5 g cell paste per liter culture.

Cell paste totaling 64 g was thawed and resuspended in NPi-10 (5 ml/g) and supplemented with Benzonase-Nuclease (0.1 μl/ml, Millipore-Sigma), SIGMAFAST Protease Inhibitor Tablet (1 tablet per 100 ml, Millipore-Sigma), and lysozyme (1 mg/ml, Millipore-Sigma). The suspension was stirred for ∼45 min and then subjected to three rounds of 4 min of sonication, 15 s on/15 s off, 30% amplitude. During sonication, cells were placed in an ice bath and allowed to rest on ice for several minutes between rounds. Cell debris was removed by centrifugation (33,000 × *g*, 65 min, 4°C), and the clarified lysate was loaded onto a hand-poured Ni column (1 ml resin per 30 ml lysate), which had been equilibrated with 10 column volumes (CV) of NPi-10. Next, the column was washed with 5 CV of NPi-10 followed by 5 CV of NPi-20. Finally, 6HMBPWgkA was eluted from the column with 10 CV of NPi-500. The elution was then concentrated ∼5× using an Amicon Ultra Centrifugal Filter (Millipore-Sigma, Membrane NMWL, 30 kDa) and exchanged into cleavage buffer by gel filtration using Sephadex G-25 resin. The 6HMBPWgkA solution collected off the G-25 column was then supplemented with HRV-3C protease and incubated at 4°C for 16 h.

The tagless WgkA peptide was purified by preparative HPLC. Following centrifugation and syringe filtration, the reaction mixture was manually injected onto a Phenomenex Jupiter 300-Å C_18_ column (5 μm, 250 × 15 mm), which had been equilibrated with 5% MeCN (in water plus 0.1% formic acid). A gradient of 5 to 25% MeCN over 10 min at a flow rate of 12 ml/min was used to elute the peptide, which came off at 21 to 23% MeCN. The relevant fractions were collected and lyophilized.

The lyophilized material was resuspended in ∼1 ml cleavage buffer and supplemented with CaCl_2_ at a final concentration of 20 mM and trypsin (2,000 ng per 1 ml). The cleavage reaction was incubated at 37°C for 16 h and then resolved by HPLC purification using a Phenomenex Luna Omega Polar C_18_ 100-Å (5-μm, 4.6 × 100 mm) column operating at 0.5 ml/min. An isocratic program of 100% H_2_O (plus 0.1% FA) over 15 min was applied to elute tryglysin. A broad peak corresponding to tryglysin was collected from 8 to 10 min. The identity of the purified sample was confirmed by HPLC−Q-TOF-MS.

### Preparation of synthetic tryglysin B.

S. mutans tryglysin was prepared analogously to its *S. ferus* counterpart according to the methods described above with minor modifications. For purification of HRV-3C-cleaved WgkA by preparative HPLC, a gradient of 5 to 35% MeCN over 15 min was used for to elute the peptide which came off at 25 to 27% MeCN. The trypsin reaction was resolved using a Phenomenex Synergi Fusion RP 80 Å (4-μm, 4.6 × 100 mm) column with a 100% H_2_O (plus 0.1% FA) isocratic step. Tryglysin eluted from 5 to 6 min.

### Identification and quantification of tryglysin A from *S. ferus*.

S. ferus DSM 20646 was grown overnight in Todd-Hewitt broth plus 0.2% yeast extract (5% CO_2_, 37°C) to an OD_600_ ≈ 2. Part (11.25 ml) of the overnight culture was centrifuged, and the cell pellet was resuspended in 225 ml of chemically defined media (79) (5% inoculum). After 6 h (OD_600_ ≈ 0.5) 50-ml aliquots were collected every 3 h up to 15 h (OD_600_ ≈ 2). Each aliquot was centrifuged and filtered, and supernatants were passed through a 20-ml Waters Oasis HLB cartridge (Waters). Compounds were eluted from the HLB cartridge with methanol, these methanolic elutions were then evaporated to dryness, and compounds were resuspended in 100 μl of 0.1% FA for LC-MS using an Agilent 6540 UHD accurate-mass quadrupole time of- flight (Q-TOF) mass spectrometer with a Phenomenex Luna Omega Polar C_18_ 100-Å (1.6 μm, 150 × 2.1 mm). Compounds were eluted using an initial 3.5-min isocratic step running 100% H_2_O with 0.1% FA, followed by a gradient to 100% acetonitrile (ACN) (plus 0.1% FA) over 16.5 min. The resulting MS data were inspected manually and additionally by using the Find Compounds by Formula feature in the Agilent MassHunter Qualitative Analysis software (version B.06, Agilent) to search for possible tryglysin matches. All ions detected from *S. ferus* extracts that were possible matches to any of the predicted tryglysin mature products based on their accurate mass and isotopic distribution were subsequently targeted for MS/MS analysis. MS/MS spectra from candidate ions were then manually inspected to identify ions with fragment ions consistent with the predicted tryglysin product match. For the time course experiment, S. ferus DSM 20646 was cultured as described above with 225 ml of CDM, and 1-ml aliquots were collected at 3, 5, 7, 8, 11, 13, 15, 17, 24, 30, 36, and 48 h. These aliquots were centrifuged, and 3 μl of supernatant were directly injected into the LC-MS using LC-MS using an Agilent 6546 UHD accurate-mass quadrupole time of flight (Q-TOF) mass spectrometer with a Phenomenex Luna Omega Polar C_18_ 100-Å column (1.6 μm, 150 × 2.1 mm). Tryglysin A was eluted using an initial 2-min isocratic step running 100% H_2_O (plus 0.1% FA) followed by a gradient to 50% ACN with 0.1% FA over 4.5 min. Concentrations of tryglysin A in supernatants were calculated from ion counts of tryglysin A [M + 2H]^2+^, observed as *m/z*: 412.1957, in samples using a standard curve with synthetic tryglysin A.

### Luciferase assays with chromosomal reporters and P*_sigX_* plasmid reporter.

For luciferase assays with *luxAB* reporters placed at the UA159 *galK* chromosomal locus and the P*_sigX_-luxAB* plasmid reporter, prestored glycerol stocks of S. mutans strains at −80°C were inoculated into THY broth and incubated overnight at 37°C in an atmosphere of 5% CO_2_. CDM was prepared and prewarmed at 37°C in an atmosphere of 5% CO_2_ overnight. The next morning, strains were transferred to sterile 15-ml conical tubes and centrifuged at room temperature at 4,000 × *g* for 10 min. Strains were resuspended in 1 ml prewarmed CDM, transferred to microcentrifuge tubes, and centrifuged again at room temperature at 14,000 × *g* for 5 min. Supernatant was discarded, and strains were resuspended in 1 ml fresh prewarmed CDM. Resuspended strains were then inoculated into 6 ml prewarmed CDM at 1:200 dilution. If required, at 1 h postinoculation, synthetic SHP (ETIIIIGGG) or reverse SHP (GGGIIIITE) was added to the media at 100 nM, 1 μM, or 10 μM concentrations. At each time point, strains were monitored by measuring OD_600_ with an GENESYS 30 Vis spectrophotometer (Thermo-Fisher) and by conducting luciferase measurements. Luciferase measurements were conducted as follows: at each time point, 100-μl aliquots from each strain and condition were transferred to an opaque 96-well plate. Samples were exposed to decyl aldehyde (Sigma-Aldrich) fumes for 1 min and counts per second (CPS) were measured using a Veritas microplate luminometer (Turner Biosystems). Relative light units (RLU) were calculated by normalizing CPS to OD_600_. For experiments to test whether the WGK peptide had autoinducing activity, at 1 h postinoculation, 100 nM tryglysin B or 100 nM tryglysin A was added in place of SHP peptide. OD_600_ and luciferase measurements were conducted as described above. For luciferase assays to test for XIP-SHP cross talk, at 1 hour postinoculation, 1 μM XIP (GLDWWSL) was added in place of SHP peptide. OD_600_ and luciferase measurements were conducted as described above. Data from experiments were plotted and analyzed using Graph Pad Prism 8.0.0 (GraphPad Software).

### Luciferase assays with the P*_wgkA_* plasmid-based reporter.

For luciferase assays with P*_wgk_-luxAB* plasmid-based reporter, prestored glycerol stocks of S. mutans strains at −80°C were inoculated into THY broth and incubated overnight at 37°C in an atmosphere of 5% CO_2_. CDM was prepared and prewarmed at 37°C in an atmosphere of 5% CO_2_ overnight. Overnight cultures were diluted 1:100 into prewarmed CDM and incubated at 37°C. Upon reaching an OD_600_ of 0.15, 180 μl of each culture was transferred to a 96-well clear bottom plate with 18 μl of fresh prewarmed CDM and 10 μl SHP or an equivalent volume of DMSO control. Decyl aldehyde was provided as a 1% solution in mineral oil (Sigma-Aldrich) and added to the spaces outside of each well. The plate was covered with a surfactant-treated lid, sealed, and incubated in a microplate reader (Synergy 2; BioTek) at 37°C with intermittent shaking. OD_600_ and luminescence measurements (CPS) were collected every 30 min for 10 h. Relative light units were calculated by normalizing CPS to OD_600_. Data from experiments were plotted and analyzed using Graph Pad Prism 8.0.0.

### Growth curves of strains.

For physiological analysis of strains, S. mutans strains were inoculated into THY broth from frozen glycerol stocks and incubated overnight at 37°C in an atmosphere of 5% CO_2_. The next morning, cells were washed, resuspended, and diluted into fresh prewarmed CDM as described above in the “Luciferase assays with chromosomal reporters” section. Growth was monitored every hour with a GENESYS 30 Vis spectrophotomer. Resulting data were plotted using Graph Pad Prism 8.0.0. Doubling times were calculated in Graph Pad Prism using a nonlinear regression analysis with exponential (Malthusian) growth equation. Doubling times of strains were calculated for OD_600_s from 0.05 to 0.65, we considered these ODs to be the exponential range of growth for S. mutans.

### Phase-contrast microscopy of live cells exposed to tryglysins.

For microscopic analysis of live cells exposed to tryglysins, S. mutans strains were inoculated and grown as described above in the “Luciferase assays with chromosomal reporters” section. If required, at 1 h postinoculation, 100 nM tryglysin A or B was added to the media. For microscopic analysis of samples, 2 μl of live cells was taken at 2 h, 5 h, and 7 h after the addition of tryglysins and examined using an Olympus CKX53 inverted microscope with a 40× LCACHN-iPC infinity objective (LCACHN40XIPC; numerical aperture, 0.55) connected to a SC50 camera (Olympus). Images were processed using Olympus Stream Image Analysis software (Olympus).

### Phase-contrast microscopy of fixed cells exposed to tryglysin A.

For microscopic analysis of fixed cells exposed to tryglysin A, S. mutans strains were inoculated and grown as described above in the “Luciferase assays with chromosomal reporters” section. If required, at 1 h postinoculation, 100 nM tryglysin A was added to the media. At 2 h, 5 h, and 7 h after the addition of tryglysin A, 6 ml of cells per strain and condition were harvested by centrifugation at 4,000 × *g* for 10 min. Supernatant was discarded, and cells were resuspended in 1 ml of fresh CDM. Resuspended cells were dropped into 10 ml of ice-cold 80% methanol and incubated for 1 h at room temperature. Afterwards, 800 μl of 4% paraformaldehyde was added to each methanol cell suspension. The mixture was incubated at room temperature for 5 min, and cells were spun down at 2,500 × *g* for 10 min. Supernatant was discarded, and cells were resuspended in 200 to 800 μl chilled GTE buffer (50 mM glucose, 20 mM Tris-HCl [pH 7.5], 1 mM EDTA). Cells were kept at 4°C overnight, and then 2 to 5 μl of cells in GTE buffer were placed on a microscope slide. Prepared slides were examined using an Olympus BX51 fluorescence microscope with a 100× UPLFLN objective (UPLFLN 100XO2; numerical aperture, 1.3) connected to an Olympus DP72 Single Chip color charge-coupled-device (CCD) camera. Images were processed using ImageJ 1.52i (80). For analysis of chain length upon tryglysin A treatment, a minimum of 100 cells were manually examined per strain and condition. Cells were classified as being in chains greater than three per chain or equal to or lesser than three per chain. The percentage of cells belonging to either of these categories was calculated as the number of cells in the category/total number of cells. Resulting data were plotted and analyzed using Graph Pad Prism 8.0.0. For statistical analysis, data were analyzed via a one-way analysis of variance (ANOVA) with Dunnet’s multiple comparison posttest in Graph Pad Prism.

### Experiments to examine the effect of tryglysins on growth of bacterial strains.

For experiments to examine the effect of tryglysins, bacterial strains were grown from prestored glycerol stocks at −80°C and inoculated into THY broth. Strains were incubated overnight at 37°C in an atmosphere of 5% CO_2_, and the next morning, they were washed and resuspended in fresh, prewarmed CDM as described above in the “Luciferase assays with chromosomal reporters” section. At 1 h postinoculation, strains were exposed to the indicated concentrations of tryglysin A or B. The following concentrations were used in experiments: 10 nM, 100 nM, or 1 μM of the indicated tryglysin. To monitor growth, OD_600_ was measured using a GENESYS 30 Vis spectrophotometer every hour. Data were plotted using Graph Pad Prism 8.0.0. and are summarized in Table 3.

### Experiment to determine whether SHP can induce immunity in S. mutans UA159 to tryglysin B.

For experiments to determine whether SHP peptide can induce immunity in S. mutans UA159 to tryglysin B, S. mutans UA159 was grown overnight, washed, and resuspended in CDM as outlined above in the “Luciferase assays with chromosomal reporters” section. At 1 h postinoculation, no SHP, 1 μM SHP, or 1 μM reverse SHP peptide was added to media. Strains were placed back in growth conditions, and at 2 h after the addition of SHP or reverse SHP, 100 nM tryglysin B was added to appropriate tubes. Growth was then monitored every hour after addition. To monitor growth, OD_600_ was measured using a GENESYS 30 Vis spectrophotometer. Data were plotted using Graph Pad Prism 8.0.0.

### UV light inactivation of tryglysin A and growth curve to determine activity.

To inactivate tryglysin A by UV light, aliquots of tryglysin A were placed in a completely dark chamber and exposed to short-wavelength UV light for 1 h, 2 h, or 4 h. After UV inactivation, aliquots were placed at −80°C until use. To determine whether UV inactivation altered activity of tryglysin A, S. mutans UA159 was grown overnight, washed, and resuspended in CDM as outlined above in the “Luciferase assays with chromosomal reporters” section. Replicate tubes were inoculated into prewarmed CDM and 1 h after inoculation of either 100 or 500 nM fresh tryglysin A, tryglysin A inactivated by UV light for 1, 2, or 4 h was added to the tubes. To monitor growth, OD_600_ was measured using a GENESYS 30 Vis spectrophotometer every hour, and data were plotted using Graph Pad Prism 8.0.0.

### CFU/ml assay to determine whether tryglysins are bacteriostatic or bactericidal for S. mutans.

To perform CFU/milliliter assays, S. mutans was grown overnight, washed, and resuspended in CDM as outlined above in the “Luciferase assays with chromosomal reporters” section, with the exception that replicate tubes for harvest at each time point for both no-tryglysin and tryglysin-treated conditions were inoculated from bacterial cultures resuspended overnight. At 1 h postinoculation, 100 nM tryglysin A or B was added to the appropriate tubes. At this time point (0 h) and at 1, 2, 4, 6, 8 and 24 h after tryglysin addition, a set of no-tryglysin and tryglysin-treated tubes were harvested. At each time point, 100 μl from each tube in a set was harvested and diluted 1:10 successively in fresh THY. Dilutions from 10^−3^ to 10^−6^ (depending on the time point) were plated as follows: for each dilution, 10 μl was plated in triplicate on THY agar plates and allowed to drip vertically down the plate. This allowed for the spread of S. mutans colonies for later CFU/ml counting. After each dilution plating, plates were placed at 37°C and incubated for 48 h. We noted that colonies were too small to count after 1 day of incubation, and accurate counts required at least 2 days of incubation. After 2 days of incubation, colonies were counted for each time point, replicate, and condition, and the number of CFU/milliliter was calculated. Drip plates were considered to have accurate counts if colonies were between 10 and 150 colonies per drip. Plates with colonies in excess of 150 were considered “too many to count.” Resulting data were plotted and analyzed using Graph Pad Prism 8.0.0. Statistical analysis was performed using a one-way ANOVA with Sidak’s multiple comparison posttest on Graph Pad Prism.

### Isolation of RNA from bacterial cultures.

For isolation of RNA, prestored glycerol stocks of S. mutans strains at −80°C were inoculated into CDM supplemented with appropriate antibiotics and incubated overnight at 37°C in an atmosphere of 5% CO_2_. The next morning, bacterial cultures were diluted 1:50 (OD_600_ ≈ 0.05) into prewarmed CDM. Once an OD_600_ of 0.3 was reached, 10 μM SHP or an equivalent amount of DMSO control as added to strains. Following 2.5 h of incubation, which corresponded to an OD_600_ ≈ 0.6, cultures were harvested by centrifugation. Supernatant was discarded, and cell pellets were resuspended in 1 ml RNALater (Ambion) and incubated at room temperature for 10 min. Samples were transferred to microcentrifuge tubes and centrifuged at 14,000 × *g* for 1 min. Supernatant was discarded, and cell pellets were stored at −80°C before proceeding to RNA purification. Total RNA was extracted using the Ambion RiboPure Bacteria kit (Ambion) as follows. Unless otherwise indicated, all following reagents were supplied by the kit. After storage at −80°C, cell pellets were thawed and resuspended in 350 μl RNAWIZ. Pellets were transferred to sterile microcentrifuge tubes containing RNase-free zirconia beads and subsequently lysed using a Minibeadbeater (BioSpec) set to homogenize for 10 min. Lysed cells were centrifuged at 14,000 × *g* for 5 min. After centrifugation, the green and cloudy aqueous phases of the resulting supernatant were transferred to sterile, RNase-free microcentrifuge tubes. To each sample, 0.2 volumes of chloroform were added, and samples were shaken vigorously for 30 s. Samples were then incubated at room temperature for 10 min and subsequently centrifuged at 14,000 × *g* for 5 min. The top clear phase of the samples was carefully transferred to a new microcentrifuge tube, and 0.5 volumes of 100% ethanol were added to each sample. Samples were mixed thoroughly and transferred to RNA-free filter cartridges in collection tubes and centrifuged at 14,000 × *g* for 1 min. Flow through was discarded, 700 μl of wash solution 1 was added to filter cartridges, and samples were centrifuged at 14,000 × *g* for 1 min. Flow through was discarded, and samples were washed with 500 μl of wash solution 2/3 and centrifuged at 14,000 × *g* for 1 min. This step was repeated, and then filters were centrifuged at 14,000 × *g* for 1 min to remove excess wash solution from cartridges. Elution solution was preheated at 99°C, and to each filter 25 μl of heated elution solution was added. Filters transferred to fresh microcentrifuge tubes and centrifuged at 14,000 × *g* for 1 min. Filters were discarded, and samples were treated with DNase 1 (Thermo-Fisher) as follows. To each sample, 1/9th the volume of 10× DNase buffer (Thermo-Fisher) and 4 μl DNase to each RNA sample were added. Samples were incubated at 37°C for 30 min. To inactivate DNase, DNase inactivation reagent (Thermo-Fisher) equivalent to 20% of the volume of the RNA sample was added, and tubes were vortexed briefly. Samples were incubated at room temperature for 2 min and subsequently centrifuged at 14,000 × *g* for 1 min to pellet DNase inactivation reagent. DNase-treated RNA samples were carefully transferred to new RNase-free microcentrifuge tubes. RNA sample purity and quantity were assessed using a Nanodrop spectrophotometer ND-1000 (Thermo-Fisher).

### Preparation of cDNA libraries for RNA sequencing.

cDNA libraries for RNA sequencing were generated from 100 ng of RNA from each sample using the KAPA Stranded RNA-seq Library Preparation kit for Illumina Platforms (Roche) according to the manufacturer’s instructions. Briefly, rRNA-depleted RNA was fragmented by heating fragmentation buffer at 94°C for 6 min. First-strand cDNA was synthesized using random primers followed by second-strand synthesis and marking and the addition of a poly(A) tail. Illumina adaptors were ligated to the fragments, and the subsequent library was subjected to two rounds of cleanup using magnetic beads for DNA purification. This was followed by library amplification for 10 PCR cycles and an additional magnetic-bead cleanup step. All libraries were then sent to the Genome Research Core at University of Illinois at Chicago for quality control and quantification on the Tapestation 2200 instrument (Agilent). At least 20 μl of 50 nM libraries was sent to the University of Chicago Genomics Facility for sequencing via an Illumina HiSeq4000 (Illumina).

### RNA sequencing analysis.

Raw sequencing data obtained from the University of Chicago Genomics Facility were analyzed by the Research Informatics Core at the University of Illinois at Chicago. Data were aligned to the S. mutans UA159 genome using the BWA MEM algorithm (81), and gene expression was quantified using featureCounts (82). Gene expression was normalized to counts per million sequences, and the differential expression analysis was performed on raw counts using edgeR (83). The false-discovery rate (FDR) correction was used to adjust *P* values for multiple testing. For volcano plot graphs, Graph Pad Prism 8.0.0 was used to construct volcano plots of final analyzed RNA-seq data. Heatmaps of RNA-seq data were generated using the ComplexHeatmap package in RStudio (84, 85). To create heatmaps of RNA-seq data, log_2_ fold change (log_2_FC) values from the RNA sequencing data were mapped. In the heatmaps generated, increasing red color indicates increased expression, whereas increasing blue indicated decreased expression. For genome-wide changes in gene expression, log_2_FC values for each gene in the genome were in descending order of locus tag number.

### Preparation of cDNA for qRT-PCR and qRT-PCR experiments.

Total RNA from S. mutans strains was used to prepare cDNA using the Superscript III first-strand synthesis system (Thermo-Fisher) according to the manufacturer’s instruction, including treatment with RNase H. Gene-specific primers used for cDNA synthesis (GS116, *gyrA*; GS130, *wgkB*; GS258, *irvA*; GS260, *gbpC*; GS268, *comEA*; GS270, *comYA*; GS274, *irvR*) and quantitative reverse transcription-PCR (qRT-PCR) primers and are listed in Table S1. cDNA resulting from gene-specific primers was diluted 1:100 and used for qRT-PCR. qRT-PCR was performed using 1× Fast SYBR green master mix (Thermo-Fisher) with gene-specific primers according to the manufacturer’s instructions and performed on a CFX Connect real-time PCR detection system (Bio-Rad). As a reference gene, SMU_1114 (*gyrA*) was used, as this gene has been previously reported not to have differential expression during growth (86, 87). Nontemplate controls were included to confirm the absence of primer-dimer formation. All samples were run in triplicate biological and technical replicates on a single plate. Relative gene expression was determined using the 2−ΔΔCT method (88). Data were plotted using Graph Pad Prism 8.0.0.

### 5′ RACE and determination of the *wgk* operon transcription start site.

Total RNA was isolated as detailed above in the section “Isolation of RNA from bacterial cultures” with the exception that bacterial cultures were induced with 1 μM SHP 1 h after inoculation 1:200 into prewarmed CDM from CDM-washed THY overnight cultures, and cultures were harvested for RNA extraction at an OD_600_ of ∼0.6 to 1. After isolation of total RNA, cDNA synthesis of the *wgk* transcript and template switching with the NEB Template Switching RT Enzyme Mix (catalog no. M0466S; NEB) were performed as specified by the manufacturer for 5′ RACE using the Template Switching RT Enzyme Mix, a gene-specific primer for the *wgk* operon (BR315) and the recommended Template Switching Oligo (TSO) (BR311). PCR amplification of the 5′ region of the *wgk* transcript was performed using Phusion High Fidelity DNA polymerase (catalog no. M0530S: NEB) using a TSO-specific primer (BR312) and another *wgk* operon-specific primer (BR316). The recommended PCR cycling condition was performed with the following changes for use of Phusion: initial denaturation was set at 95°C for 2 min, initial annealing and extension were set at 60°C, secondary annealing and extension were set at 60°C, and final annealing was set at 60°C. All other parameters were used per manufacturer’s instructions for amplification of the 5′ region of transcripts. Resulting PCR products were sequenced via Sanger sequencing at the University of Illinois at Chicago Genome Research Core, and sequences were examined for where the TSO primer sequence and *wgk* operon sequence met, indicating the transcription start site for *wgk*.

10.1128/mBio.02688-20.10 TEXT S1 Supplemental references. Download Text S1, DOCX file, 0.02 MB. Copyright © 2021 Rued et al. 2021 Rued et al. https://creativecommons.org/licenses/by/4.0/ This content is distributed under the terms of the Creative Commons Attribution 4.0 International license.
