# Erratum for Rued et al., “Quorum Sensing in Streptococcus mutans Regulates Production of Tryglysin, a Novel RaS-RiPP Antimicrobial Compound”

**DOI:** 10.1128/mBio.02560-21

**Published:** 2021-10-05

**Authors:** Britta E. Rued, Brett C. Covington, Leah B. Bushin, Gabriella Szewczyk, Irina Laczkovich, Mohammad R. Seyedsayamdost, Michael J. Federle

**Affiliations:** a Department of Pharmaceutical Sciences, University of Illinois at Chicago, Chicago, Illinois, USA; b Department of Chemistry, Princeton University, Princeton, New Jersey, USA

## ERRATUM

Volume 12, no. 2, e02688-20, 2021, https://doi:10.1128/mBio.02688-20. The first error regards Table S1. Unfortunately, we provided an incorrect version of the table that contains incorrect references. The table has been replaced online. Second, we regret not including NIH training grant information for Britta Rued. Britta Rued was supported by the grant while she was collecting data and generating the manuscript. The grant identification number is 5K12 GM139186/NIGMS/NIH.

