# Supplementary material for: Quorum Sensing in Streptococcus mutans Regulates Production of Tryglysin, a Novel RaS-RiPP Antimicrobial Compound
Source: mBio. 2021 Mar 16;12(2):e02688-20. doi: 10.1128/mBio.02688-20 (PMC8092268; doi:10.1128/mBio.02688-20)
Supplement: TABLE S1 [file mBio.02688-20-st001.pdf]

TABLE S1. Bacterial strains, plasmids, and primers used in this study.[a]

Strain/Plasmid	Description	Antibiotic Resistance [b]	Reference
***S. mutans* strains**			
BRSM01	*Streptococcus mutans* UA159 wild-type reference strain	NR	(1)
BRSM05	UA159 Δ*pdrA*::*spec* (ΔSMU_1509::*spec*)	$Spec^R$	This study
BRSM08	UA159 Δ$wgkAB_{N\text{-}term}$::*spec*[c]	$Spec^R$	This study
BRSM38	UA159 Δ*galK*::P_c-*erm*	Erm^R	This study
BRSM49	UA159 *galK*::P_{wgkA}-*luxAB*-P_c-*erm*	Erm^R	This study
BRSM64	UA159 Δ*rgg*::*spec* *galK*::P_{wgkA}-*luxAB*-P_c-*erm* (BRSM05 transformed with *galK*::P_{wgkA}-*luxAB*-P_c-*erm* from BRSM49)	$Spec^R$, Erm^R	This study
BRSM69	UA159 *galK*::P_{shp}-luxAB-P_c*erm*	Erm^R	This study
BRSM73	UA159 Δ*rgg*::*spec* *galK*::P_{shp}-luxAB-P_c-*erm* (BRSM05 transformed with *galK*::P_{shp}-luxAB-P_c-*erm* from BRSM69)	$Spec^R$, Erm^R	This study
BRSM85	UA159 pLZ12Spec (UA159 transformed with pLZ12Spec)	$Spec^R$	This study
GS23	UA159 Δ*pptAB*::*aphA3* pGS101 (JCC263 transformed with pGS101)	Km^R, Erm^R	This study
GS105	UA159 Δ*oppD*::*cat* pJC301 pGS101 (JCC298 was electroporated with pJC301 and pGS101)	Cm^R, $Spec^R$, Erm^R	This study
JCC263	UA159 Δ*pptAB*::*aphA3*	Km^R	(21)
JCC298	UA159 Δ*oppD*::*cat* pLZ12Spec (BRSM85 was transformed with Δ*oppD*::*cat*)	Cm^R, $Spec^R$	This study
MW37	UA159 pWAR307 (UA159 transformed with pWAR307)	Cm^R	This study
**Other bacterial strains**			
A909	*Streptococcus agalactiae* A909 wild-type isolate	NR	(22)
ATCC 10556	*Streptococcus sanguinis* ATCC 10556 wild-type isolate, also known as SK1	NR	(23)
ATCC 11454	*Lactococcus lactis* ATCC 11454 wild-type isolate	NR	(24)

BH10c	*Escherchia coli* derivative of DH10B	NR	(3)
BL21(DE3)	*Escherchia coli* strain for recombinant plasmid expression	NR	NEB
BL21(DE3) + pRSFDuet-1_6HMBP*wgkA_wgkBC*_DSM20646 + pDB1282	BL21(DE3) deriviative for co-expression of 6HMBPWgkA, WgkB and WgkC from *S. ferus*	Km^R, Amp^R	This study
E. coli BL21(DE3) + pRSFDuet-pRSFDuet-1_6HMBP*wgk*A_*wgkB*-rbs-*wgkC*_UA159 + pDB1282	B21(DE3) deriviative for co-expression of 6HMBPWgkA, WgkB and WgkC from *S. mutans*	Km^R, Amp^R	This study
CCUG 31611	*Streptococcus mitis* CCUG 31611 wild-type isolate, also known as NCTC 12261	NR	(25)
D39	*Streptococcus pneumoniae* D39 wild-type isolate, from M. E. Winkler	NR	(26)
DH5α	*Escherchia coli* strain for cloning	NR	NEB
DSM 20646	*Streptococcus ferus* DSM 20646 wild-type isolate	NR	(6)
JB1	*Streptococcus bovis* JB1 wild-type isolate	NR	(27)
NZ131	*Streptococcus pyogenes* NZ131 wild-type isolate	NR	(2)
S. gordonii Challis	*Streptococcus gordonii* Challis Bt wild-type isolate	NR	(28)
S. oralis 108	*Streptococcus oralis* 108 wild-type isolate, from L. Tao	NR	(29)
V583	*Enterococcus faecalis* V583 wild-type isolate	Van^R	(30)
**Plasmids**[d]			
pBAD_6HMBPP_HTFLAG	Source of 6HMBP tag	Amp^R	Seyedsayamdost Lab
pDB1282	Vector for *isc* operon expression	Amp^R	(31)
pEVP3	Template plasmid for P_{syncat} chloramphenicol cassette	Cm^R	(32)
pGS101	pWAR303 derivative with P_{wgkA} in front of *luxAB*	Erm^R	This study
pGS103	pWAR303 derivative with P_{shp} in front of *luxAB*	Erm^R	This study
pJC300	pLZ12Spec derivative with P_{syncat} chloramphenicol cassette	Cm^R, $Spec^R$	This study
pJC301	pJC300 derivative, amplified by inverse PCR to leave P_{syncat} in front of multiple cloning site of plasmid	$Spec^R$	This study

pLZ12Spec	Shuttle vector encoding spectinomycin resistance; pWV01 origin	Spec^R	(33)
pRSFDuet-1	Co-expression vector	Km^R	Novagen
pRSFDuet-1_6HMBP	pRSFDuet-1 modified with a 6HMBP tag coding sequence	Km^R	This study
pRSFDuet-1_6HMBP*wgkA*_*wgkBC*_DSM20646	Co-expression of 6HMBPWgkA, WgkB and WgkC	Km^R	This study
pRSFDuet-1_6HMBP*wgkA*_*wgkB*-rbs-*wgkC*_UA159	Co-expression of 6HMBPWgkA, WgkB and WgkC	Km^R	This study
pWAR303	pFED761 derviative with *luxAB* fragment	Erm^R	(34)
pWAR307	pJC156 derivative with P_{sigX} in front of *luxAB*	Cm^R	(34)

Primers used for strain construction[e]

Primer	Sequence (5' to 3')[f]	Template	RE Site	Amplicon or Plasmid Product
For construction of BRSM05 (Δ*pdrA*::*spec*)				
GS111	TGGGTATGATTTGCTCTCTGCAGGA	UA159	NotI	5' upstream of *pdrA*
GS112	AAAA**GCGGCCGC**CAATAGTACCTCAATTGTCTATATGGG			
GS105	AAAA**GCGGCCGC**CATTAGGATCCAGATCTTCCTTCAGGTTATG	pLZ12 Spec	NotI, SalI	Spec cassette
GS106	GCGTG**GTCGAC**GCAAGGGTTTATTGTTTTCTAAAATCTGAT			
GS113	AAAA**GTCGAC**TAATATGAAATTGTGTATATGGGAT	UA159	SalI	3' downstream of *pdrA*
GS114	AAATAGACGAAATAGCAGCTCCACGAATT			
For construction of BRSM08 (Δ*wgkAB*$_{N-term}$::*spec*)				
GS136	GCACGTAAGAGCAACAATCTAG	UA159	NotI	5' upstream of *wgkA*
GS137	AAAA**GCGGCCGC**AGTATTTACCTCCTTGATTTGATAG			
GS105	AAAA**GCGGCCGC**CATTAGGATCCAGATCTTCCTTCAGGTTATG	pLZ12 Spec	NotI, SalI	Spec cassette
GS106	GCGTG**GTCGAC**GCAAGGGTTTATTGTTTTCTAAAATCTGAT			
GS138	AAAA**GTCGAC**TAAAACAAGGTCTGTAGGTCATTG	UA159	SalI	3' downstream of *wgkA*
GS139	GCTCCAAGAATACCTCCAAATG			
For construction of BRSM38 (Δ*galK*::P_c-*erm*)				

BR94	AGGATATCAACAGCAGTACGGCCC	UA159	5' upstream of *galK*
BR95	ATCCATTAAAAATCAAACGGATCCTATTTCCTTTACTTTTGTTATACTGAAATGAG		
BR96	CTCATTTCAGTATAACAAAAGTAAAGGAAATAGGATCCGTTTGATTTTTAATGGATA	P_c-*erm* cassette[g]	P_c-*erm* cassette
BR97	ATCTAGTAATGCTGTCATCGTTTTATTTCCTCCCGTTAAATAATAGATAACTATTA		
BR98	TAGTTATCTATTATTTAACGGGAGGAAATAAAACGATGACAGCATTACTAGATACT	UA159	3' downstream of *galK*
BR99	CAGTGTGATGCGGTGTATCA		
For construction of BRSM49 (*galK*::P_{wgkA}-*luxAB*-P_c-*erm*)			
BR94	AGGATATCAACAGCAGTACGGCCC	UA159	5' upstream of *galK*
BR192	CTGCTACTGATTTCCTTTACTTTTGTTATACTG		
BR193	GTAAAGGAAATCAGTAGCAGCAATGAAATTG	pGS101	P_{wgkA}-*luxAB*
BR97	ATCTAGTAATGCTGTCATCGTTTTATTTCCTCCCGTTAAATAATAGATAACTATTA		
BR98	TAGTTATCTATTATTTAACGGGAGGAAATAAAACGATGACAGCATTACTAGATACT	BRSM38	P_c-*erm* + 3' downstream of *galK*
BR99	CAGTGTGATGCGGTGTATCA		
For construction of BRSM69 (*galK*::P_{shp}-luxAB-P_c-*erm*)			
BR94	AGGATATCAACAGCAGTACGGCCC	UA159	5' upstream of *galK*
BR190	GACTGAATATTTTCCTTTACTTTTGTTATACTG		
BR191	GTAAAGGAAAATATTCAGTCATCAATAGTACCTC	pGS103	P_{shp}-*luxAB*
BR97	ATCTAGTAATGCTGTCATCGTTTTATTTCCTCCCGTTAAATAATAGATAACTATTA		
BR98	TAGTTATCTATTATTTAACGGGAGGAAATAAAACGATGACAGCATTACTAGATACT	BRSM38	P_c-*erm* + 3' downstream of *galK*
BR99	CAGTGTGATGCGGTGTATCA		

For construction of JCC298 (Δ*oppD*::cat)				
GS309	GCGTG**GCGGCCGC**TCTTTCTTACAAGATGCTTGGCGA	UA159	NotI	5’ upstream of *oppD*
GS310	GCGTG**ATCGAT**AACATCGCGAATAGCTTTTA		ClaI	
GS311	GCGTG**ATCGAT**GATGAAAATTTGTTTGATTT	pEVP3	ClaI	Cat cassette
GS312	GCGTG**GTCGAC**TTATAAAAGCCAGTCATTAG		SalI	
GS313	GCGTG**GTCGAC**ATGCTGCTACTTGGCTGTTAGATGA	UA159	SalI	3’ downstream of *oppD*
GS314	GCGTG**CTCGAG**AGTACAGTTAAAATAGGACT		XhoI	
For construction of pGS101 (pWAR303 P_{wgk})				
GS203	AAAA**CTGCAG**TACCTCCTTGATTTGATAGAATTA	UA159	PstI	P_{wgk}
GS204	AAAA**GTCGAC**TTTAGTGTTCCTAAAACAACTAAG		SalI	
For construction of pGS103 (pWAR303 P_{shp})				
GS206	AAAA**GTCGAC**ATATTCAGTCATCAATAGTACCTC	UA159	SalI	P_{shp}
GS207	AAAA**CTGCAG**TTCTCCTCACTCACTTTTCTTAT		PstI	
For construction of pJC300 (pLZ12Spec with P_{syncat}-cat cassette)				
JC208	CATG**GAATTC**ATTTGTTTGATTTTTAATGG	pEVP3	EcoRI	P_{syncat}-cat cassette
JC412	CATG**GAATTC**GATGGGTTCCGAGGCTC			
For construction of pJC301 (pJC300 P_{syncat})				
JC413	CATGGCGGCCGCATTAGGATCCAGATCTTCCTTCAGGTTATGACCATC	pJC300		pJC300 P_{syncat} without cat cassette
JC414	CATGGCGGCCGCCCTCCTAAATTTTTATCTAAAGTG			
For construction of pRSFDuet-1_6HMBP*wgkA*_*wgkBC*_DSM20646				
*wgkA*_DSM20646_F	AGTTCTGTTTCAGGGTCCGGAATTCGGATCCATGTCACCTAAAAAAGAGTTTAATGCTCC	DSM 20646		Cloning *wgkA* into pRSFDuet-1_6HMBP at MCS1
*wgkA*_DSM20646_R	ATGCGGCCGCAAGCTTGTCGACCTGCAGTTAATGCTTACCCCAACTATTAACTTTAGTTG			
*wgkBC*_DSM20646_F	GTTAAGTATAAGAAGGAGATATACATATGAGAGATTATTCGCCATATCCATTATTAGTAG	DSM 20646		Cloning *wgkBC* into pRSFDuet-1_6HMBP at MCS2
*wgkBC*_DSM20646_R	GGTTTCTTTACCAGACTCGAGTTATTTCTCCCTAAAAGGTTTTAACTGTAAGTATATATC			
For construction of pRSFDuet-pRSFDuet-1_6HMBP*wgk*A_*wgkB*-rbs-*wgkC*_UA159				

*wgkA*_UA159_F	ATGATT**GGATCC**ATGCTGACCAAAAAAGAGTTCAGCGTGC	UA159	BamHI	Cloning *wgk*A into pRSFDuet-1_6HMBP at MCS1
*wgkA*_UA159_R	TGAAGA**CTGCAG**TTAATGTTTGCCCCAGCAGTTCACTTTGG		PstI	
*wgkB*_UA159_F	ACGGTG**CATATG**CGCGACTATTCTCCGTATCCG	UA159	NdeI	Cloning *wgkB*-rbs-*C* into pRSFDuet-1_6HMBP at MCS2
wgkB-rbs_UA159	CATGGTATATCTCCTTATTAATTATCCTCGTCATAGAAGCATGCCAG	UA159		
rbs-*wgkC*_UA159	GACGAGGATAATTAATAAGGAGATATACCATGAAAAACCATTTCACCATCAAAGGCAAGC	UA159		Cloning *wgkB*-rbs-*C* into pRSFDuet-1_6HMBP at MCS2
*wgkC*_UA159_R	GGGGGG**CTCGAG**TCATAAAGCTTCGCGAAACGGTTTCAGATACAG	UA159	XhoI	

Primers for qRT-PCR and 5' RACE

Primer	Sequence (5' to 3')	Description
BR311	GCTAATCATTGCAAGCAGTGGTATCAACGCAGAGTACATGGG	Template switching oligonucleotide (TSO) for 5' RACE
BR312	CATTGCAAGCAGTGGTATCAAC	TSO annealing primer
BR315	CTAGTGCTTACCCCAACAGTTT	Reverse primer for *wgkA*
BR316	CCCCAACAGTTTACCTTAGTTG	Reverse primer for *wgkA*
GS116	ATTGTTGCTCGGGCTCTTCC	Forward primer for *gyrA* (SMU_1114)
GS117	TGCGGCTTGTCAGGAGTAAC	Reverse primer for *gyrA* (SMU_1114)
GS130	GATGTCTGTTGAGAAGCTGGAT	Forward primer for *wgkB* (SMU_1508c)
GS131	TCTAATAAACGGTTCACCTCCA	Reverse primer for *wgkB* (SMU_1508c)
GS258	CCCTCAACACACTCTGCTAAGCT	Forward primer for *irvA* (SMU_1397c)
GS259	CCAAATCATTGGCCAGTTGAA	Reverse primer for *irvA* (SMU_1397c)
GS260	AATTCTGATACTGTTGCAGCACCTA	Forward primer for *gbpC* (SMU_1396)
GS261	TTCTGTTGCAGCCGGTTCT	Reverse primer for *gbpC* (SMU_1396)
GS268	AGGAACAATCCCTTCAGGTAACC	Forward primer for *comEA* (SMU_625)
GS269	CAGTCGTCTGCGTCTTCTTCTG	Reverse primer for *comEA* (SMU_625)
GS270	CTTTTTTCTGGACGTCACGATTT	Forward primer for *comYA* (SMU_1987)

GS271	TCGCCCCTTGATTTCATT TAA	Reverse primer for *comYA* (SMU_1987)
GS274	CTAGCTGAGAGCGGAAT GAAA	Forward primer for *irvR* (SMU_1398)
GS275	TTTGGCAAACTCTTTCCC TTAAC	Reverse primer for *irvR* (SMU_1398)

[a]Strains were constructed as described in *Materials and Methods*.

[b]Antibiotic resistance markers: NR, no resistance markers; Amp^R, ampicillin resistance; $Spec^R$, spectinomycin resistance; Erm^R, erythromycin resistance; Km^R, kanamycin resistance; Cm^R, chloramphenicol resistance; Van^R, vancomycin resistance.

[c]Δ*wgkAB*$_{N\text{-}term}$::*spec* refers to a deletion of the coding region of *wgkA* and the first 992 nucleotides of *wgkB*, replaced by a spectinomycin cassette. See *Materials and Methods* for additional details.

[d]All plasmids were constructed and/or propagated in the *E. coli* BH10c, DH5α, or BL21(DE3) strains.

[e]Genomic DNA of UA159 or indicated plasmid DNA was used as template for PCR or restriction nucleotide reactions.

[f]Bolded and underlined nucleotides indicate engineered restriction sites, if used.

[g]P_c-*erm* cassette was derived from fusion of the synthetic P_c promoter sequence from Land et. al, 2011 (35) with the erythromycin resistance cassette from pWAR303.
