# Supplementary figures and images for: Quorum Sensing in Streptococcus mutans Regulates Production of Tryglysin, a Novel RaS-RiPP Antimicrobial Compound
Source: mBio. 2021 Mar 16;12(2):e02688-20. doi: 10.1128/mBio.02688-20 (PMC8092268; doi:10.1128/mBio.02688-20)

Figure S1

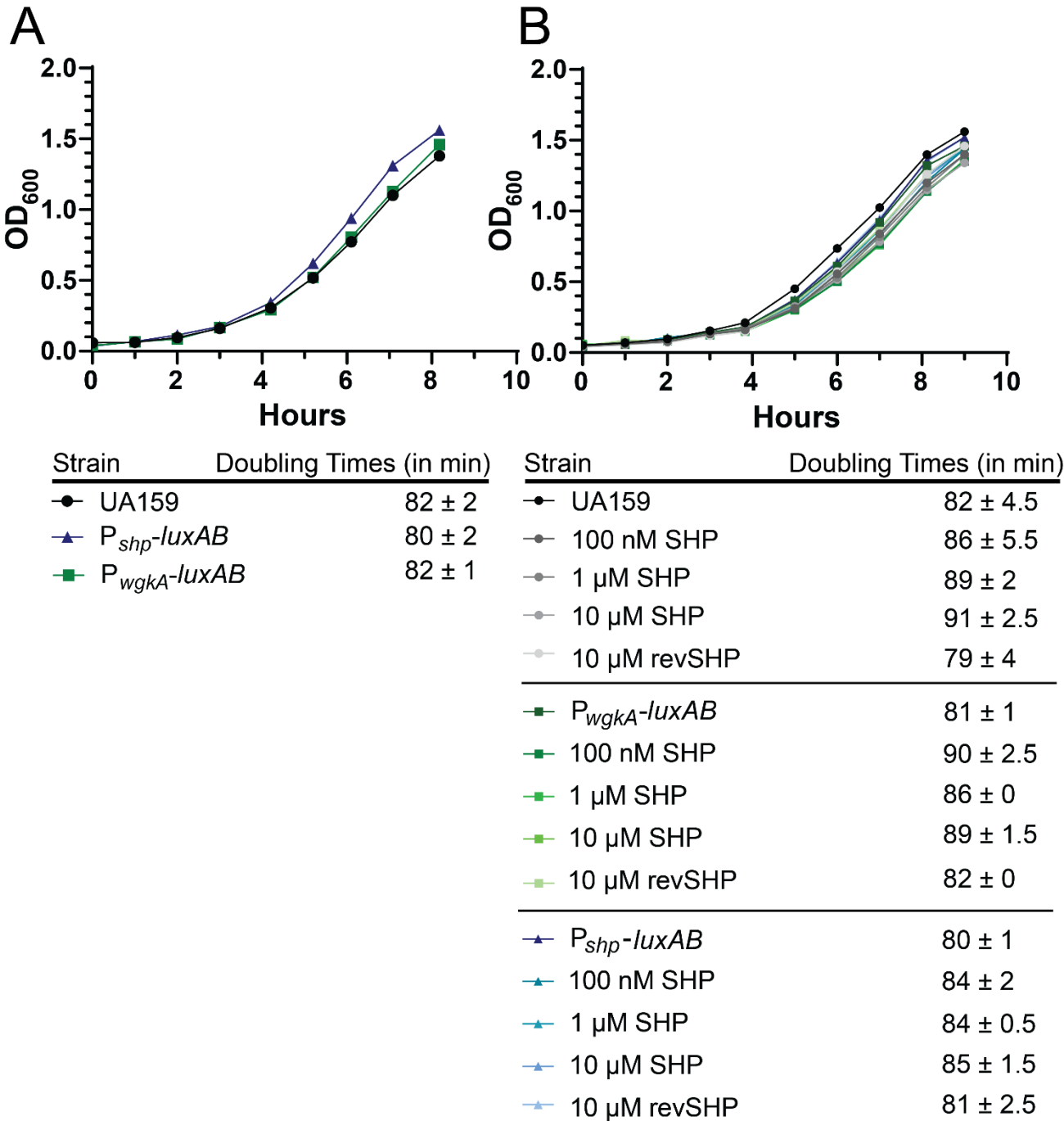

Supplement: FIG S1 [file mBio.02688-20-sf001.pdf]

Figure S2

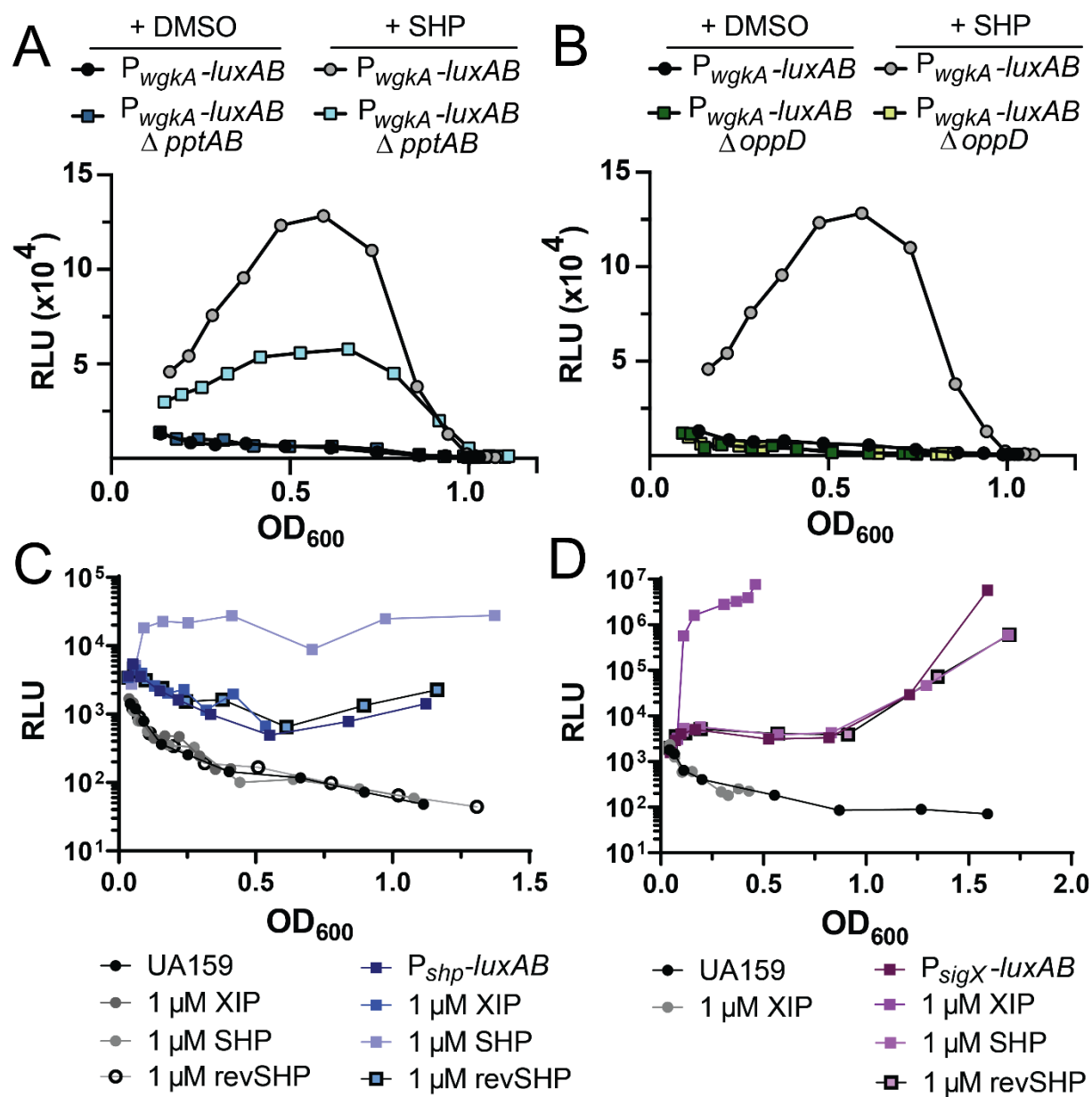

Supplement: FIG S2 [file mBio.02688-20-sf002.pdf]

Figure S3

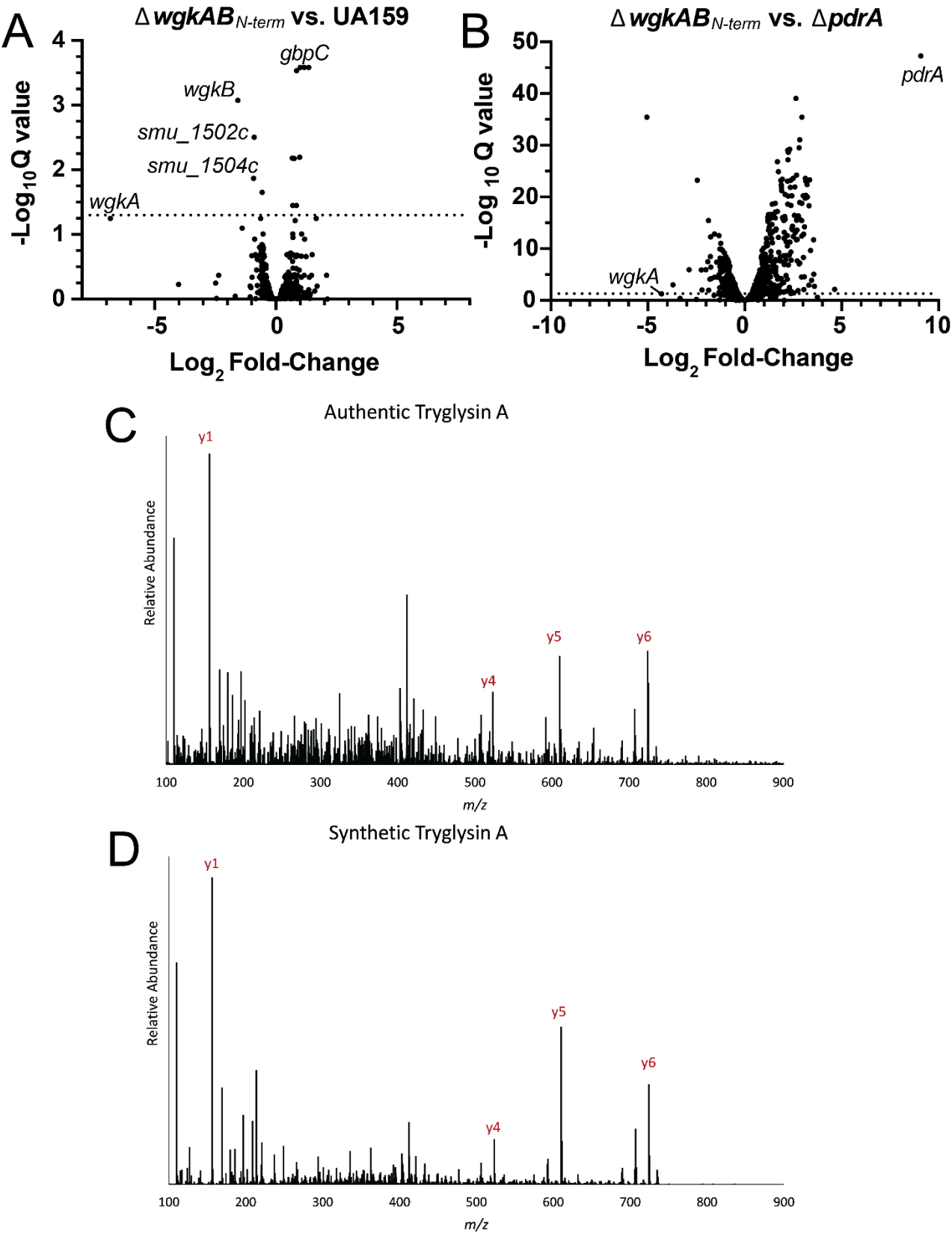

Supplement: FIG S3 [file mBio.02688-20-sf003.pdf]

Figure S6

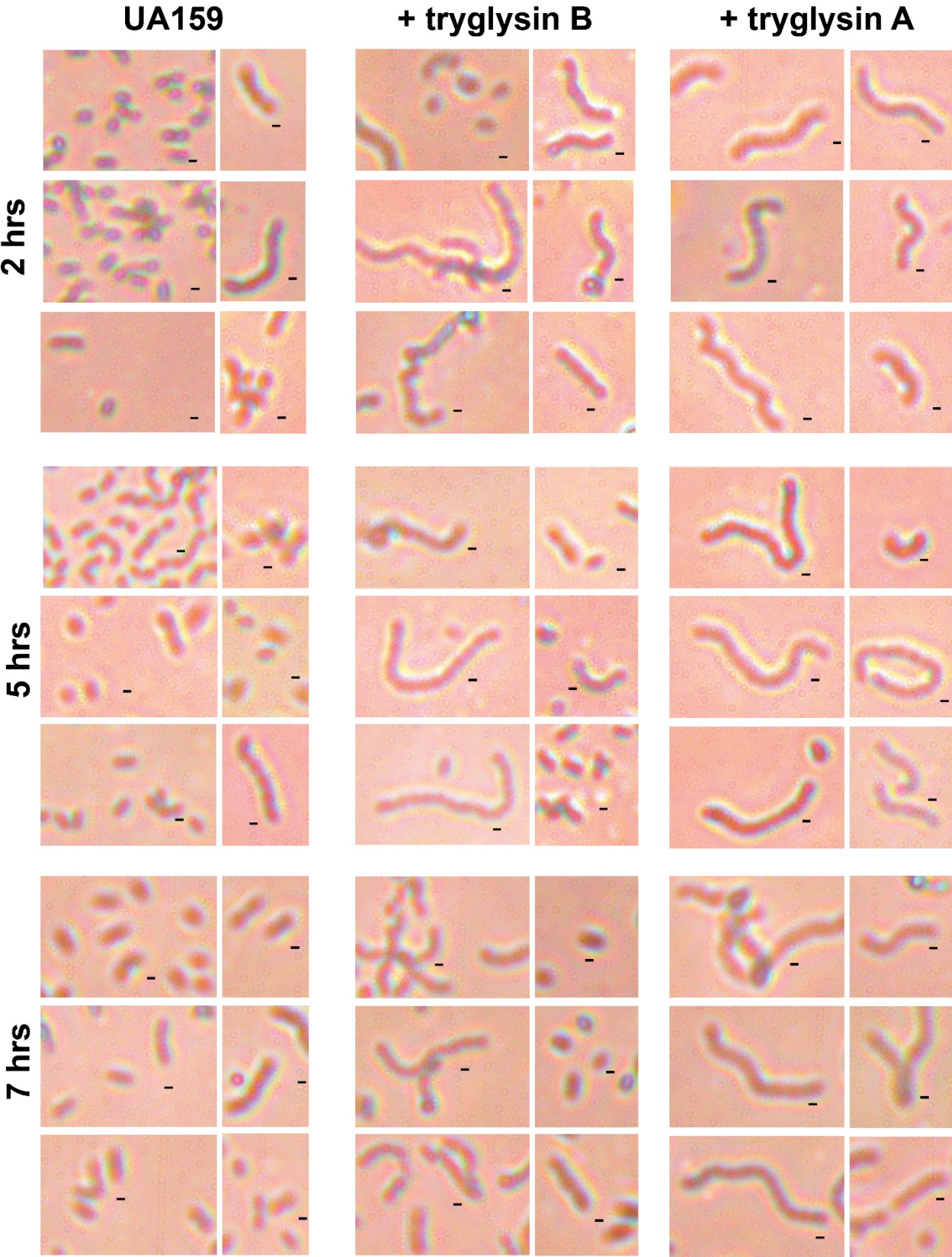

Supplement: FIG S6 [file mBio.02688-20-sf006.pdf]

Figure S4

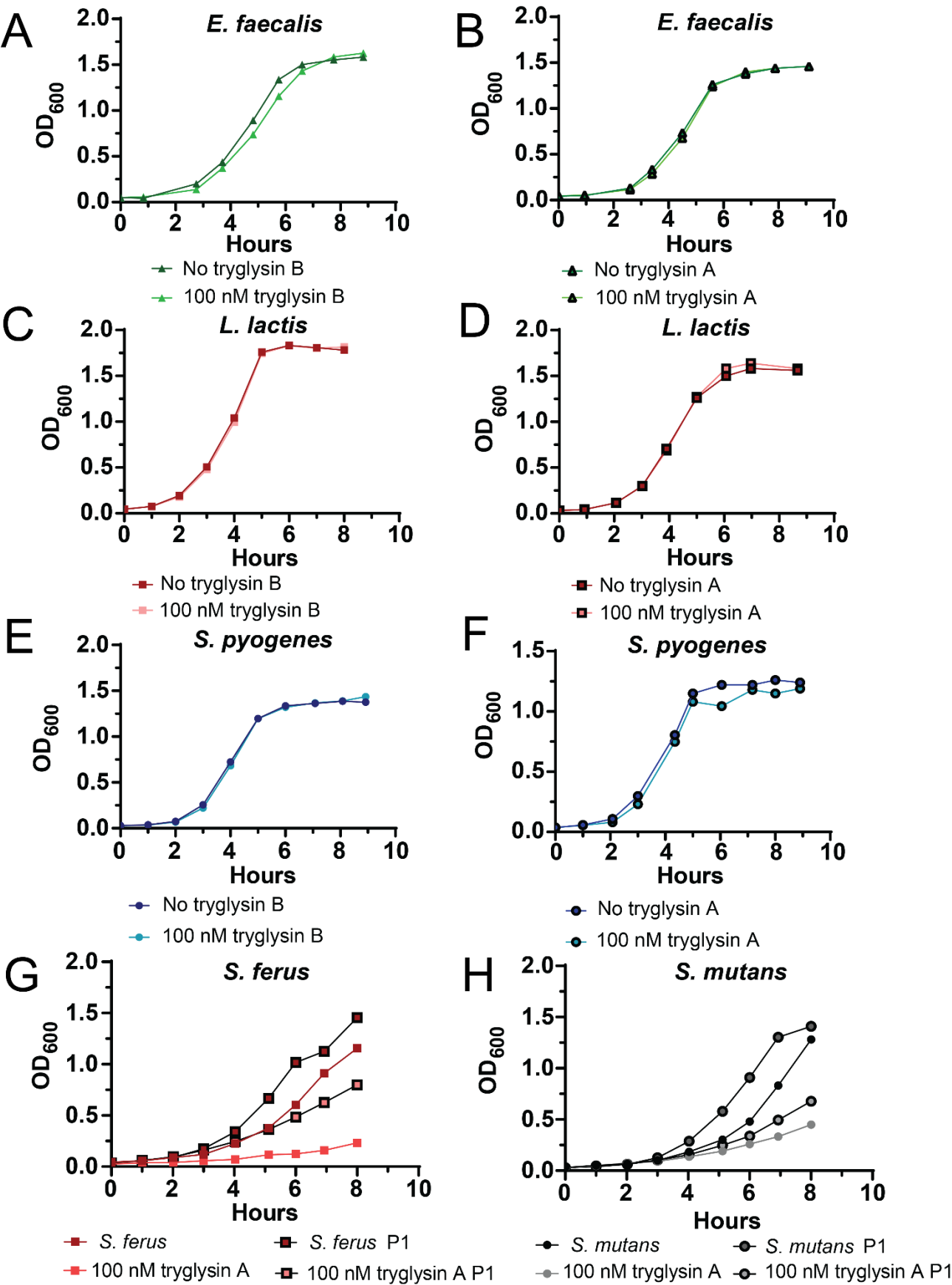

Supplement: FIG S4 [file mBio.02688-20-sf004.pdf]

Figure S5

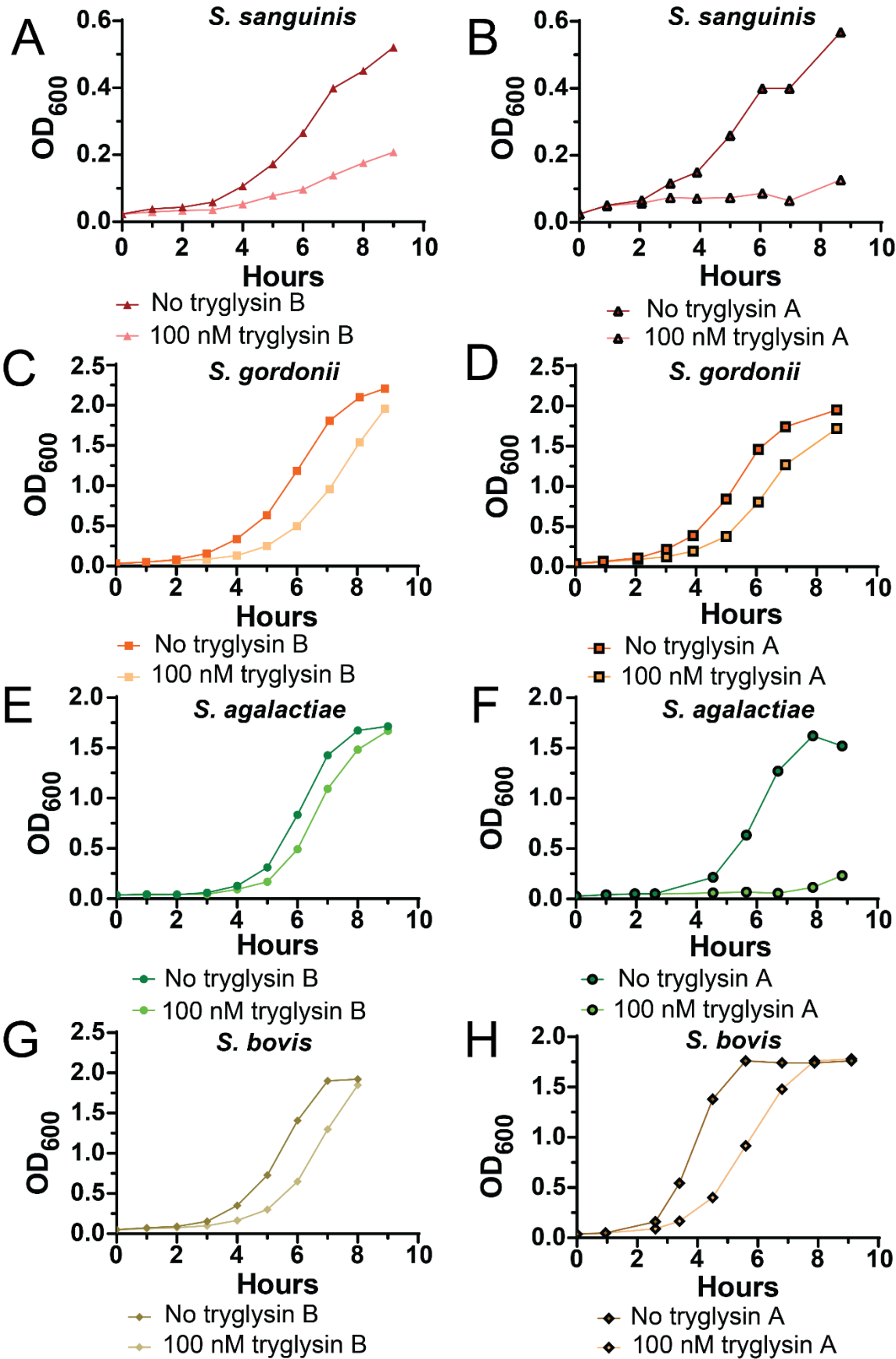

Supplement: FIG S5 [file mBio.02688-20-sf005.pdf]
