# Supplementary material for: Quorum Sensing in Streptococcus mutans Regulates Production of Tryglysin, a Novel RaS-RiPP Antimicrobial Compound
Source: mBio. 2021 Mar 16;12(2):e02688-20. doi: 10.1128/mBio.02688-20 (PMC8092268; doi:10.1128/mBio.02688-20)
Supplement: TABLE S2 [file mBio.02688-20-st002.pdf]

Table S2A. HR-MS and HR-MS/MS data for authentic tryglysin A from *S. ferus* DSM 20646.

Ion	Calculated m/z	Observed m/z	Δppm	Sequence
b_2^{+1}	214.1186	214.1182	1.9	VN
y_1^{+1}	156.0768	156.0776	5.1	H
y_4^{+1}	523.2412	523.2409	0.6	WGKH
y_5^{+1}	610.2732	610.2743	1.8	SWGKH
y_6^{+1}	724.3161	724.3216	7.6	NSWGKH
MH^{+1}	823.3846	823.3868	2.7	VNSWGKH

Table S2B. HR-MS data for synthetic tryglysin A and B.

Strain	*S. ferus* DSM 20646			*S. mutans* UA159		
Sequence	VNSWGKH			VNCWGKH		
Ion	Calc	Obs	Δppm	Calc	Obs	Δppm
$[M+1H]^{1+}$	823.38456	823.38595	1.7	839.36172	839.35906	3.2
$[M+2H]^{2+}$	412.19592	412.19676	2.0	420.18450	420.18453	0.1
$[M+3H]^{3+}$	275.13304	275.13486	6.6	280.45876	280.45928	1.9

Table S2C. HR-MS/MS data for synthetic tryglysin A.

Ion	Calculated m/z	Observed m/z	Δppm	Sequence
b_2^{+1}	214.1186	214.1163	10.7	VN
y_1^{+1}	156.0768	156.07368	20.0	H
y_4^{+1}	523.2412	523.2393	3.6	WGKH
y_5^{+1}	610.2732	610.2705	4.4	SWGKH
y_6^{+1}	724.3161	724.3134	3.7	NSWGKH
$MH-H_2O^{+1}$	805.3740	805.3694	5.7	VNSWGKH
$MH-NH_3^{+1}$	806.3580	806.3561	2.4	VNSWGKH
MH^{+1}	823.3846	823.3814	3.9	VNSWGKH

Table S2D. HR-MS data for modified HRV-3C-cleaved WgkA peptides, intermediates toward the preparation of synthetic tryglysin A and B (see Materials and Methods).

Strain	*S. ferus* DSM 20646			*S. mutans* UA159		
Sequence	GPEFGS-MSPKKEFNAPKTTKVNSWGKH			GPEFGS-MLTKKEFSVPKTTKVNCWGKH		
Ion	Calc	Obs	Δppm	Calc	Obs	Δppm
$[M+2H]^{2+}$	1493.23743	1493.23747	0.0	1516.75969	1516.76051	0.5
$[M+3H]^{3+}$	995.82738	995.82938	2.0	1011.50888	1011.50965	0.8
$[M+4H]^{4+}$	747.12235	747.12758	7.0	758.88348	758.88480	1.7
$[M+5H]^{5+}$	597.89934	597.90667	12.3	607.30824	607.30998	2.9

Table S2E. HR-MS/MS data for synthetic tryglysin B.

Ion	Calculated m/z	Observed m/z	Δppm	Sequence
b_2^{+1}	214.1186	214.1179	3.3	VN
y_1^{+1}	156.0768	156.0758	6.4	H
y_4^{+1}	523.2412	523.2329	15.9	WGKH
y_5^{+1}	626.2504	626.2507	0.5	CWGKH
y_6^{+1}	740.2933	740.2923	1.4	NCWGKH
MH^{+2}	420.1845	420.1851	1.4	VNCWGKH
