# Supplementary material for: Quorum Sensing in Streptococcus mutans Regulates Production of Tryglysin, a Novel RaS-RiPP Antimicrobial Compound
Source: mBio. 2021 Mar 16;12(2):e02688-20. doi: 10.1128/mBio.02688-20 (PMC8092268; doi:10.1128/mBio.02688-20)
Supplement: TEXT S1 [file mBio.02688-20-s0001.docx]

**SUPPLEMENTAL TEXT**

Quorum sensing in *Streptococcus mutans* regulates production of tryglysin, a novel RaS-RiPP antimicrobial compound.

.

Britta E. Rued,^a^ Brett C. Covington,^b^ Leah B. Bushin,^b^ Gabriella Szewczyk,^a^***** Irina Laczkovich,^a^ Mohammad R. Seyedsayamdost,^b^# Michael J. Federle^a^#.

^a^Department of Pharmaceutical Sciences. University of Illinois at Chicago, Chicago, Illinois, USA

^b^Department of Chemistry, Princeton University, Princeton, New Jersey, USA

Running Head: Quorum sensing regulates an antimicrobial peptide in *S. mutans*

#Address correspondence to Michael J. Federle ([mfederle@uic.edu](mailto:mfederle@uic.edu)) or to Mohammad R. Seyedsayamdost (mrseyed@princeton.edu).

*Present address: College of Dentistry, University of Illinois at Chicago, Chicago, Illinois, USA.

**SUPPLEMENTARY REFERENCES**

1. Ajdić D, McShan WM, McLaughlin RE, Savić G, Chang J, Carson MB, Primeaux C, Tian R, Kenton S, Jia H, Lin S, Qian Y, Li S, Zhu H, Najar F, Lai H, White J, Roe BA, Ferretti JJ. 2002. Genome sequence of *Streptococcus mutans* UA159, a cariogenic dental pathogen. Proc Natl Acad Sci U S A 99:14434–9.

2. Chang JC, Federle MJ. 2016. PptAB exports Rgg quorum-sensing peptides in Streptococcus. PLoS One 11:e0168461.

3. Tettelin H, Masignani V, Cieslewicz MJ, Donati C, Medini D, Ward NL, Angiuoli S V., Crabtree J, Jones AL, Durkin AS, DeBoy RT, Davidsen TM, Mora M, Scarselli M, Margarit Y Ros I, Peterson JD, Hauser CR, Sundaram JP, Nelson WC, Madupu R, Brinkac LM, Dodson RJ, Rosovitz MJ, Sullivan SA, Daugherty SC, Haft DH, Selengut J, Gwinn ML, Zhou L, Zafar N, Khouri H, Radune D, Dimitrov G, Watkins K, O’Connor KJB, Smith S, Utterback TR, White O, Rubens CE, Grandi G, Madoff LC, Kasper DL, Telford JL, Wessels MR, Rappuoli R, Fraser CM. 2005. Genome analysis of multiple pathogenic isolates of *Streptococcus agalactiae*: Implications for the microbial “pan-genome.” Proc Natl Acad Sci U S A 102:13950–5.

4. Gong K, Wen DY, Ouyang T, Rao AT, Herzberg MC. 1995. Platelet receptors for the *Streptococcus sanguis* adhesin and aggregation- associated antigens are distinguished by anti-idiotypical monoclonal antibodies. Infect Immun 63:3628–33.

5. Steen MT, Yong Joon Chung, Hansen JN. 1991. Characterization of the nisin gene as part of a polycistronic operon in the chromosome of *Lactococcus lactis* ATCC 11454. Appl Environ Microbiol 57:1181–8.

6. Howell-Adams B, Steven Seifert H. 2000. Molecular models accounting for the gene conversion reactions mediating gonococcal pilin antigenic variation. Mol Microbiol 37:1146–58.

7. Sánchez-Beato AR, García E, López R, García JL. 1997. Identification and characterization of IS1381, a new insertion sequence in *Streptococcus pneumoniae.* J Bacteriol 179:2459–63.

8. Lanie JA, Ng WL, Kazmierczak KM, Andrzejewski TM, Davidsen TM, Wayne KJ, Tettelin H, Glass JI, Winkler ME. 2007. Genome sequence of Avery’s virulent serotype 2 strain D39 of *Streptococcus pneumoniae* and comparison with that of unencapsulated laboratory strain R6. J Bacteriol 189:38–51.

9. Bushin LB, Clark KA, Pelczer I, Seyedsayamdost MR. 2018. Charting an Unexplored Streptococcal Biosynthetic Landscape Reveals a Unique Peptide Cyclization Motif. J Am Chem Soc 140:17674–17684.

10. Benahmed FH, Gopinath GR, Harbottle H, Cotta MA, Luo Y, Henderson C, Teri P, Soppet D, Rasmussen M, Whitehead TR, Davidson M. 2014. Draft genome sequences of *Streptococcus bovis* strains ATCC 33317 and JB1. Genome Announc 2:e01012-14.

11. Chang JC, LaSarre B, Jimenez JC, Aggarwal C, Federle MJ. 2011. Two group a streptococcal peptide pheromones act through opposing rgg regulators to control biofilm development. PLoS Pathog 7:e1002190.

12. Sulavik MC, Tardif G, Clewell DB. 1992. Identification of a gene, *rgg*, which regulates expression of glucosyltransferase and influences the Spp phenotype of *Streptococcus gordonii* Challis. J Bacteriol 174:3577–86.

13. Xu H, Sobue T, Bertolini M, Thompson A, Dongari-Bagtzoglou A. 2016. *Streptococcus oralis* and *Candida albicans* Synergistically Activate μ-Calpain to Degrade E-cadherin from Oral Epithelial Junctions. J Infect Dis 214:925–34.

14. Paulsen IT, Banerjei L, Hyers GSA, Nelson KE, Seshadri R, Read TD, Fouts DE, Eisen JA, Gill SR, Heidelberg JF, Tettelin H, Dodson RJ, Umayam L, Brinkac L, Beanan M, Daugherty S, DeBoy RT, Durkin S, Kolonay J, Madupu R, Nelson W, Vamathevan J, Tran B, Upton J, Hansen T, Shetty J, Khouri H, Utterback T, Radune D, Ketchum KA, Dougherty BA, Fraser CM. 2003. Role of mobile DNA in the evolution of vancomycin-resistant *Enterococcus faecalis*. Science (80 ) 299:2071–4.

15. Johnson DC, Unciuleac MC, Dean DR. 2006. Controlled expression and functional analysis of iron-sulfur cluster biosynthetic components within *Azotobacter vinelandii*. J Bacteriol 188:7551–61.

16. Pestova E V., Morrison DA. 1998. Isolation and characterization of three *Streptococcus pneumoniae* transformation-specific loci by use of a *lacZ r*eporter insertion vector. J Bacteriol 180:2701–10.

17. Husmann LK, Scott JR, Lindahl G, Stenberg L. 1995. Expression of the Arp protein, a member of the M protein family, is not sufficient to inhibit phagocytosis of *Streptococcus pyogenes*. Infect Immun 63:345–8.

18. Mashburn-Warren L, Morrison DA, Federle MJ. 2010. A novel double-tryptophan peptide pheromone is conserved in mutans and pyogenic Streptococci and Controls Competence in *Streptococcus mutans* via an Rgg regulator. Mol Microbiol 78:589–606.

19. Land AD, Winkler ME. 2011. The requirement for pneumococcal MreC and MreD is relieved by inactivation of the gene encoding PBP1a. J Bacteriol 193:4166–79.
